# Meis1 Is Required for Adult Mouse Erythropoiesis, Megakaryopoiesis and Hematopoietic Stem Cell Expansion

**DOI:** 10.1371/journal.pone.0151584

**Published:** 2016-03-17

**Authors:** Michelle Erin Miller, Patty Rosten, Madeleine E. Lemieux, Courteney Lai, R. Keith Humphries

**Affiliations:** 1 Terry Fox Laboratory, British Columbia Cancer Agency, Vancouver, British Columbia, Canada; 2 Bioinfo, Plantagenet, Ontario, Canada; 3 Department of Medicine, University of British Columbia, Vancouver, British Columbia, Canada; French Blood Institute, FRANCE

## Abstract

*Meis1* is recognized as an important transcriptional regulator in hematopoietic development and is strongly implicated in the pathogenesis of leukemia, both as a Hox transcription factor co-factor and independently. Despite the emerging recognition of *Meis1*’s importance in the context of both normal and leukemic hematopoiesis, there is not yet a full understanding of *Meis1*’s functions and the relevant pathways and genes mediating its functions. Recently, several conditional mouse models for *Meis1* have been established. These models highlight a critical role for *Meis1* in adult mouse hematopoietic stem cells (HSCs) and implicate reactive oxygen species (ROS) as a mediator of *Meis1* function in this compartment. There are, however, several reported differences between these studies in terms of downstream progenitor populations impacted and effectors of function. In this study, we describe further characterization of a conditional knockout model based on mice carrying a loxP-flanked exon 8 of *Meis1* which we crossed onto the inducible Cre localization/expression strains, B6;129-*Gt(ROSA)26Sor*^*tm1(Cre/ERT)Nat*^/J or B6.Cg-Tg(Mx1-Cre)1Cgn/J. Findings obtained from these two inducible Meis1 knockout models confirm and extend previous reports of the essential role of *Meis1* in adult HSC maintenance and expansion and provide new evidence that highlights key roles of Meis1 in both megakaryopoiesis and erythropoiesis. Gene expression analyses point to a number of candidate genes involved in Meis1’s role in hematopoiesis. Our data additionally support recent evidence of a role of *Meis1* in ROS regulation.

## Introduction

*Myeloid ecotropic virus insertion site 1 (Meis1)* was first described in 1995 as a common viral integration site in the BXH-2 murine model of myeloid leukemogenesis [1]. Since its discovery, *Meis1* has attracted interest by virtue of its intimate association with *Hox* genes in the development of leukemia in mouse models and frequent co-expression with *HOX* genes in human acute myeloid leukemia samples [1–3]. As a *Hox* co-factor, Meis1 confers specificity to Hox targets. In the hematopoietic system high levels of *Meis1* expression largely mirror that of various *Hox* genes through development and hematopoietic differentiation. Loss of *Meis1* is embryonic lethal [4, 5] at d11.5–14.5 days post coitum (dpc) due to abnormal vascularization, haemorrhage from a lack of platelets and an absence of functional hematopoietic stem cells (HSCs).

Embryonic lethality has limited our understanding of *Meis1* in the adult organism until the recent development of conditional *Meis1* knockout animals. Two such models for the study of *Meis1* function have recently been described. Two groups independently have exploited a conditional knockout model developed by the group of Drs. Nancy Jenkins and Neal Copeland on a C57BL/6 J background in which a loxP-flanked exon 8 allele of *Meis1* (*Meis1*^*flfl*^) allows for Cre-induced deletion. The resultant truncated transcript lacks coding regions for the critical homeodomain and C- terminal region. Unnisa *et al*. crossed the *Meis1*^*fl/fl*^ mice onto the *Rosa26CreER* mice for tamoxifen (4-OHT) inducible deletion [6], whereas, Kocabas *et al*. [7] exploited this model using two lineage specific Cre-recombinase expressing mouse lines (*Scl-Cre-ER*^*T*^ and *αMHC-Cre* [7, 8]). Independently, Ariki *et al*. derived a similar lox-P flanked exon 8 of *Meis1* and studied this on the interferon inducible *Mx1-Cre* background [9]. Combined, these studies have described a clear hematopoietic stem cell (HSC) deficit in adult mice lacking *Meis1*, although the impact on downstream progenitors and conclusions regarding underpinning mechanisms have been somewhat discordant between the models. For example, while all groups describe a loss of HSC numbers and quiescence, only Kocabas *et al*. were able to demonstrate an increase in apoptosis in comparison to Unnisa *et al*. and Ariki *et al*. who found no differences in Annexin V staining of purified HSC subsets in the absence of *Meis1* [6, 7, 9]. While both Unnisa *et al*. and Kocabas *et al*. have data suggesting a role for *Hif1α*, Ariki *et al*. were not able to replicate this finding in their model [6, 7, 9].

Contemporary to the above studies, our group was also provided access to the *Meis1*^*fl/fl*^ mouse line developed by Drs. Jenkins and Copeland. We independently validated the *Meis1*^*fl/fl*^ allele and studied the phenotype of mice lacking *Meis1* using both the inducible expression and protein stabilization of Cre recombinase using the *Mx1-Cre* (*MxCre)* and *Rosa26CreER* (*ERTCre)* mouse models. We examined the histological, phenotypic and functional impact on HSCs and downstream progenitors and additionally quantified these deficits. Our data confirm and extend a key role for *Meis1* in primitive hematopoietic repopulating cells and, furthermore, point to critical roles in megakaryocytic and erythroid progenitor expansion *in vivo*. Additional evidence is also provided for a role of *Meis1* in reactive oxygen species (ROS) regulation. We additionally identify two putative effectors of *Meis1* function through gene expression analysis. Overall, our work using two model systems of *in vivo Meis1* deletion extends our understanding of *Meis1* function in normal hematopoiesis and highlights intriguing avenues for further investigation.

## Materials and Methods

### Mice

All mice (*Mus musculus*) were bred and maintained at the British Columbia Cancer Agency Animal Resource Centre (ARC) with all protocols approved by the University of British Columbia Animal Care Committee (Certificate: A13-0063). Male *Meis1*^*tmloxP/+*^ (*Meis1*^*fl/+*^) mice on the CD45.2 C57BL/6 J (B6) background with a loxP-flanked exon 8 of the endogenous *Meis1* allele were a generous gift from Drs N. Jenkins and N. Copeland. The derivation of these mice is described in Kocabas *et al*., and Unnisa *et al*., [6, 7]. *Meis1*^fl/+^ mice were bred with B6;129-*Gt(ROSA)26Sor*^*tm1(Cre/ERT)Nat*^/J (*ERTCre;* Gift from Dr. A. Weng) or B6.Cg-Tg(Mx1-Cre)1Cgn/J (*MxCre;* Jackson Laboratory) for inducible expression of *Cre* recombinase and subsequent deletion of *Meis1* exon 8 (*MxCre/Meis1-/-* or *ERTCre/Meis1-/-*). For transplantation assays, wild-type B6.SJL-PtprcaPeb3b/BoyJ (Pep3b, BC Cancer Agency ARC, Vancouver, BC) mice irradiated with 780 cGy using an X-ray source were used as recipients. Bone marrow (BM) from non-irradiated Pep3b mice was also used in the MEINOX family gene expression assays.

Induction of *Cre* expression was achieved in *MxCre/Meis1*^*fl/fl*^ *or* ^*fl/+*^ mice by intraperitoneal (IP) injection of 300μg polyinosinic:polycytidylic acid (PolyI:C; VWR/EMD Biosciences, Radnor, PA, USA), dissolved in phosphate buffered saline (PBS; STEMCELL Technologies Inc., Vancouver, BC, CAN) per mouse every 48 hours for 18 days. To induce nuclear localization of Cre and hence deletion of *Meis1* in *ERTCre/Meis1*^*fl/fl*^ *or* ^*fl/+*^ mice, 4-hydroxytamoxifen (4-OHT; Sigma, St. Louis, MO, USA) was first dissolved at 25mg/mL in filtered >99% ethanol (EtOH) and homogenized with a hand-held homogenizer. This was further diluted into autoclaved corn oil (Sigma) to achieve a 5mg/mL 4-OHT suspension. 1mg 4-OHT was injected IP into *ERTCre/Meis1* mice every 48 hours for 12 days to induce *Cre* expression.

Phenylhydrazine treatment is an experimental model for hemolytic anemia [10] and was used to examine the capacity of *Meis1*^*-/-*^ erythroid-progenitors to expand in response to stress. At 48-hours following the last of nine PolyI:C injections, *MxCre/Meis1*^*-/+*^ or *MxCre/Meis1*^*-/-*^ mice were given IV injections of phenylhydrazine hydrochloride (PHZ; Sigma) at 40mg/Kg. Four days later, mice were euthanized and analyzed for phenotype and CFC (colony forming cells) (described below) capacity.

For studies examining the role of ROS in *Meis1* function, Cre expression in *MxCre/Meis1* mice was induced with PolyI:C according to the protocol outlined above. Following two PolyI:C injections (4 days), mice were then also given daily subcutaneous (SQ) injections of 100mg/Kg *N*-*acetylcysteine* (NAC, Sigma) for 14 days. Mice were analyzed 5 to 7 days following the final PolyI:C/NAC injections.

In all cases, mice were housed in the BC Cancer Agency ARC, a specific-pathogen free facility. Mice were housed with their littermates at the appropriate ratio and used between 6 to 8 weeks of age, regardless of the experiment. Mice were euthanized according to ARC protocols using CO_2_ at the end of the experimental period or if the mouse became moribund. Every effort was made to foster animal well-being and minimize suffering.

### Isolation of bone marrow and peripheral blood for analysis

Peripheral blood (PB) parameters were monitored using the tail prick method and collection into heparinized capillary tubes. For PB cell count and differential, blood was transferred to ETDA-coated blood collection tubes (microtainer 365973, BD, Franklin Lakes, NJ, USA) and counts performed by a Scil Vet ABC automatic blood cell counter (Vet Novations, Viernheim, Germany). If mice were euthanized prior to blood collection, blood was collected from the heart. BM was isolated by flushing the marrow cavities of femurs, tibias and iliac crests with 2% fetal bovine serum (FBS; STEMCELL) in phosphate buffered saline (2% FBS in PBS) using an 18G to 24G needle.

### Long-term repopulating cell (LTRC) and competitive repopulating unit (CRU) assays

BM was collected in 2% FBS in PBS and nucleated cell counts performed using 3% v/v acetic acid with methylene blue. If no further manipulation (such as cell sorting) was required prior to transplantation, appropriately diluted Ly5.2+ cell suspensions were made and transplanted into recipient Ly5.1^+^ Pep3b mice irradiated at 780 cGy using an X-ray source. Whole Pep3b BM was used as helper or competitor cells as indicated in the results.

Engraftment of test cells into recipients was monitored by PB collection from the tail-vein of recipient mice at various intervals up to 16 weeks post-transplantation. Approximately 50μL of PB was collected, lysed (Pharmlyse, BD Biosciences) and incubated at 4°C with a combination of fluorochrome conjugated anti-mouse antibodies against CD45.2-FITC (anti-Ly5.2), CD4-PE, CD8-PE, B220-PE, B220-APC-Cy7, Gr1-APC-Cy7 and Mac1-APC-Cy7 (Table A in S1 File for clones and suppliers). Samples were washed and re-suspended in 2% FBS in PBS and 1ug/mL propidium iodide (PI) prior to acquisition on a modified FACSCalibur (BD with Cytek laser and digital detector upgrades, Fremont, CA, USA). Analysis of FACS data was performed using FlowJo analysis software (TreeStar, Ashland, OR, USA). Mice were scored as positive for multi-lineage engraftment if >1% of total PB nucleated cells were donor-derived Ly5.2^+^ cells and within this population, the contribution of T cells (CD4CD8^+^), B-cells (B220^+^) and myeloid cells (Gr1^+^Mac1^+^) was >1%. Long-term repopulating cell frequency and test for significant differences between groups was performed using the Extreme Limiting Dilution Analysis (ELDA) online tool (http://bioinf.wehi.edu.au/software/elda) [11].

### Histology

The sternum, spleen and, in some cases, the tibia, of mice were isolated and placed directly into 4% paraformaldehyde solution. These specimens were fixed and mounted onto slides by the Provincial Health Services Authority Histology Laboratory (Vancouver, BC, CAN). Slides were stained with hematoxylin and eosin (H&E), anti-CD45R (RA3-62B, BD Pharmingen) or anti-Ter119 antibodies (TER-119, BD Pharmingen) using the Ventana Discovery XT (Roche) machine and protocols. Both anti-CD45R and anti-Ter119 antibodies were used at a 1:50 dilution and linked to anti-rabbit HRP using a rabbit anti-rat antibody (Jackson Immuno Research) secondary at 1:500 dilution.

### Isolation and phenotypic analysis of primary marrow progenitor and mature cell populations

*MxCre/Meis1* mice were analyzed 5–7 days following the final PolyI:C injection as trials at earlier time-points yielded FACS profiles with indistinct separation between positive and negative populations. Analysis of *ERTCre/Meis1* mice was done 2–4 days following the final 4-OHT injection. Isolated BM was suspended in 2% FBS in PBS and red blood cells (RBCs) were lysed with PharmLyse (BD Biosciences) reagent according to the manufacturers instructions. A viable cell count was then performed using 0.4% trypan blue in PBS. Unless the cells were destined for a myeloid progenitor sort (common myeloid progenitor (CMP), granulocyte-monocyte progenitor (GMP) or megakaryocyte-erythroid progenitor (MEP), cells were blocked for 20 minute on ice in 5% rat sera and 1μg/1x10^6^ cells Fc receptor (FcR, also known as CD16/32), then washed with 2% FBS in PBS. Cells were then incubated with the antibody stains outlined in Table A in S1 File for 20 min on ice. Cells were then washed and re-suspended at roughly 1x10^7^ cells/mL with 3μM 4',6-diamidino-2-phenylindole (DAPI, Life Technologies, Carlsbad, CA, USA) for viability and 10μg/mL DNaseI solution (STEMCELL Technologies) to prevent clumping. Cells were then filtered through a 45μm filter prior to sorting.

An Influx II or Aria cytometer with 488nm argon, 350/405nm UV and 634nm red diode laser sources were used for cell sorting and phenotyping from primary marrow. Cells were sorted/analyzed according to the gating strategies outlined in Table B in S1 File. Annexin V staining for apoptosis and BrDU for cell cycle analyses were done according to the kit manufacturer’s instructions (BD Biosciences). Following sorting, purity was assessed by running a small fraction of the sorted sample through the cytometer. Purity of >98% was deemed to be acceptable. Analysis of FACS data was performed using FlowJo analysis software (TreeStar). Unpaired two-tailed, Student’s T-tests were performed to determined statistical significance between *Meis1*^*-/-*^ and control samples.

### Hematopoietic colony-forming cell (CFC) assays

Marrow for CFC analysis for *MxCre/Meis1* mice was collected 5–7 days after induction of deletion in order to collect samples at the time of FACS analysis to minimize the numbers of mice required for the studies and mitigate residual immunological PolyI:C effects. Isolated BM cells were suspended in 2% FBS in PBS while spleen cells were isolated by maceration through a 0.2μM screen. Nucleated cell counts were performed and cells further diluted in 2% FBS in Iscoves Modified Dulbecco Medium (2%FBS in IMDM, STEMCELL Technologies). BM or spleen cells were mixed with methylcellulose media containing cytokines to support CFU-GEMM, CFU-GM, and BFU-E colony growth (CAT: M3434, STEMCELL) according to manufacturer’s protocol at a concentration of 2x10^4^ cells per dish for BM or 2x10^5^ cells per dish for spleen. As this cocktail supports myeloid colony growth that can overwhelm the less frequent erythroid progenitors, an alternate methylcellulose media optimized for BFU-E growth (CAT: M3436, STEMCELL Technologies) was also used. For the erythroid colony cultures, 4x10^4^ BM or 4x10^5^ spleen cells per dish were plated. Cells were cultured for 10–14 days in a humidified incubator at 37°C and 5% CO_2_ and colony morphology and numbers assessed using an inverted microscope.

CFU-Mk are difficult to detect using in situ morphological assessment and are therefore more accurately enumerated by detection in collagen gels that have been dehydrated, fixed and treated with a cytochemical stain for acetylcholinesterase enzyme activity. BM was plated at a concentration 1x10^5^ cells per culture slide into collagen-based media supplemented with cytokines and lipids (CAT 4964, STEMCELL, Technologies) according to manufacturer’s instructions. Cultures were incubated at 37° 5% CO_2_ and >95% humidity for 7 days, then fixed, stained and enumerated according manufacturer’s instructions using SIGMA staining reagents.

### Long-term culture initiating cell (LTC-IC) assay

S17 stromal line cells [12] were irradiated at 2000cGy and seeded at 1.5x10^4^ cell per well into a flat-bottom tissue culture treated 96-well plate. Cultures were then seeded with un-separated BM cells at various concentrations (3x10^4^, 1.5x10^4^, 7.5x10^3^, 3.75x10^3^) from induced *ERTCre/Meis*^*+/-*^, *ERTCre/Meis*^*-/-*^, *MxCre/Meis*^*+/-*^ or *MxCre/Meis*^*-/-*^mice. Cells were prepared in mouse MyeloCult™ M5300 (STEMCELL) supplemented with freshly prepared 10^−6^ M hydrocortisone (21-hemisuccinate sodium salt)(mLTCM). Weekly one-half media changes were performed according to manufacturer’s protocol. Following 4 weeks in culture, adherent and non-adherent cells were harvested from individual wells and plated in methycellulose media supplemented with cytokines (CAT:3434, STEMCELL Technologies), then cultured as described for CFC assays. Colony numbers were counted and the wells were recorded as negative if no colony was present and positive if ≥ 1 colony was present. LTC-IC frequency and test for significant differences between groups was performed using the Extreme Limiting Dilution Analysis (ELDA) online tool (http://bioinf.wehi.edu.au/software/elda) [11].

### Southern blot analysis

Southern blot analysis to detect Cre-mediated deletion of *Meis1*^*fl/fl*^ was carried out using previously described procedures [13]. In brief, genomic DNA was extracted using DNAzol Reagent (Life Technologies). Genomic DNA (10 μg) was digested with either HindIII, EcoRI or BglII for 16 hours at 37°C and electrophoresed at 30V on a 1.0% agarose/TAE gel. Gels were treated with 0.1 M HCl for 8 minutes, rinsed with deionized H_2_O, treated with 1.5M NaCl/ 0.5N NaOH for 30 minutes and capillary transferred to ZetaProbe-GT (Bio-Rad) with 10x sodium saline citrate (SSC) for 16 hours. DNA was fixed to membranes by treating at 80°C for 1 hour. Membranes were prehybridized 2 hours at 65°C in buffer containing 0.8% skim milk, 8% Dextran Sulfate, 5X SSC, 8% Formamide, 0.8% SDS, 1.6 mM EDTA pH 8.0, and 400 μg/ml sheared and boiled Salmon Sperm DNA.

DNA probes were synthesized by PCR and gel purified prior to labeling. Probes are outlined in Table C of S1 File**.** DNA probes were labeled by random priming and incorporation with ^32^P dCTP, denatured and added to the pre-hybridization buffer with the membrane, incubated for 16 hrs at 65°C, then washed 3 times at 65°C (0.3X SSC, 0.1%SDS, 0.1% Tetra-Sodium Pyrophosphate). The hybridized membranes were then exposed to a phosphor–imaging screen for 24 hours. The phosphor screen was scanned by STORM Phosphorimager (Molecular Dynamics, Sunnyvale, CA, USA) and analyzed with ImageQuant 5.2 software (GE Lifesciences).

### RT-PCR cloning of the 5’ loxP site and truncated transcript

To confirm the positioning of the loxP sites, the products of PCR amplification using primer sets described in Table C in S1 File were TOPO TA cloned into the PCR2.1 vector (Life Technologies) and sequenced by the McGill University Sequencing Service (Montreal, Canada). The presence and position of both loxP sites were confirmed in the non-deleted mutant allele by sequence analysis, as well as the deletion of genomic sequence containing exon 8 between these loxP sites leaving a single remaining loxP site.

In order to establish if a truncated transcript was generated following *in vivo* Cre-mediated *Meis1* deletion, splenocytes were isolated from *ERTCre/Meis1*^*fl/fl*^, *ERTCre/Meis1*^*+/fl*^, and *ERTCre/Meis1*^+/+^ mice following *in vivo* Cre induction by 4-OHT. Cells were lysed in TRIZOL reagent (Life Technologies), and total RNA extracted as per recommended procedure. RNA was reverse transcribed with Superscript Vilo cDNA synthesis kit (Life Technologies) and amplified with Platinum Taq Polymerase (Life Technologies) with primers specific for *Meis1* exon7 (5’-TCCACTCGTTCAGGAGGAAC-3’) and *Meis1* exon 11(5’-TGCTGACCGTCCATTACAAA-3’). Predicted sizes of amplicons were obtained (428bp, intact exon 8; 282 bp deleted exon 8), gel purified and TOPO-TA cloned into PCR 2.1 Vector (Life Technologies). Individual clones were sequenced by McGill University Sequencing service with M13Forward and M13Reverse sequencing primers.

### Q-PCR & Q-RT-PCR

Genomic DNA purification from mouse BM and PB for <10^6^ cells was performed with PureLink^TM^ Genomic DNA Mini Kit (Life Technologies). For BM samples of >10^6^ cells, purification was performed with DNAzol Reagent (Life Technologies). DNA concentration was quantified by NanoDrop using 25 ng of DNA template per reaction. Q-PCR was performed with the 7900HT Real-Time PCR System (Applied Biosystems) with FastStart Universal SYBR Green MasterMix (Roche). Table C in S1 File lists the primer sets used for Q-PCR detection of genomic deletion of the floxed *Meis1* alleles. Primer set NDF (non-deleted floxed) is located between the loxP sequences and anchored in the 3’ loxP site and is used to detect the non-deleted floxed *Meis1* allele. Primer set DF (deleted floxed) has the forward primer 5’ of the 5’loxP site and the reverse in the targeting vector sequence 3’ of the 3’ loxP site such that only the collapsed floxed *Meis1* allele is detected. Control primers sets were used to normalize and confirm genotype. Primer set Floxed CTL uses the 3’ targeting vector sequence to detect the floxed allele regardless of collapse whereas the Exon 7 CTL set detects both wild-type and floxed *Meis1* alleles. Control *MxCre/Meis1*^*-/-*^ and *MxCre/Meis1*^*fl/fl*^ genomic DNA samples confirmed by Southern Blot analysis to be 100% and 0% collapsed, respectively, were used as calibrator samples in Relative Quantification Analysis using RQ Manager version 2.1 software (Applied Biosystems, Foster City, CA, USA).

For Q-RT-PCR, RNA from the sorted populations was extracted using RNaqueous-Micro Kit (Ambion/Life Technologies) as per manufacturers instructions due to the limiting cell numbers. Approximately 10ng of total RNA was reverse-transcribed with Superscript®Vilo cDNA Synthesis kit (Life Technologies). Prior to RT-PCR, cDNA was pre-amplified 14 cycles with Taqman PreAmp Mastermix (Applied Biosytems) with the relevant primer sets (Table D in S1 File) diluted to 1:100 for global amplification of the genes of interest. RT-PCR was performed on pre-amplified cDNA (0.125 μl per reaction) with Universal Taqman Mastermix (Applied Biosytems) and 6-FAM/ZEN/IBFQ PrimeTime® Assays (Integrated DNA Technologies, Coralville, IA, USA) on a 7900HT Fast Real-Time PCR System (Applied Biosystems). Relative quantification analysis was performed using ABL1 as endogenous control and *MxCre/Meis1*^*-/-*^ expression was compared to *MxCre/Meis1*^*-/+*^ expression for each target using RQ Manager version 1.2 Software (Applied Biosystems).

### Affymetrix mRNA array and analysis

RNA was extracted from LSK (Lin^-^Sca-1^+^ckit^+^) sorted BM from induced *MxCre*^*+*^*/Meis1*^*fl/fl*^ and *MxCre*^*+*^*/Meis1*^*+/fl*^ mice with RNAqueous-Micro Kit (Ambion) as per manufacturers recommended procedure. RNA was concentrated by ultra-centrifugation, amplified and hybridized to the Affymetrix Mouse Exon ST 1.0 Array (Affymetrix, Santa Clara CA, USA) by the BC Cancer Agency Centre for Translational and Applied Genomics (CTAG) using the Ovation Pico WTA System (NuGEN) according to manufacturer’s instructions. For quality control, 100 ng of each amplified cDNA product was tested in two separate runs by Bioanalyzer on an RNA 6000 Nano LapChip (Agilent): 1) before fragmentation and labeling (100 ng of each sample) and 2) after fragmentation and labeling. Input samples passed both the Bioanalyzer trace test for fragment size (>80% less than 200bp) and concentration and purity measurements by A320, A260 and A280 absorbance.

Affymetrix Expression Console software was used for the initial exon- and gene-based probe set signal normalization and log2 summarization. Our analysis was restricted to the well-annotated “core” exons and genes. Data were further adjusted using ComBat [14] to remove batch effects attributable to litter-to-litter variability rather than *Meis1* status. We confirmed that the loss of Meis1 exon 8 was reflected in the exon-based signal intensity. To identify differentially regulated genes, samples were compared by two-tailed T-tests with unequal variance followed by Benjamini-Hochberg correction for multiple hypothesis testing [15]. The R statistical computing environment was used for batch correction and expression analysis (R Development Core Team, 2009). Gene set enrichment analysis (GSEA) was performed using the Broad Institute platform with a false discovery rate (FDR) ≤ 0.25.

### Statistical Analysis

Unpaired, unequal variance, two-tailed, Student’s T-tests were performed to determined statistical significance between *Meis1*^*-/-*^ and control samples in Vet Analyzer samples, all semi-solid colony forming assays (CFC & CFU-Mk), RT-PCR, Q-RT-PCR and FACS analyses. Unpaired, unequal variance, two-tailed Student’s T-tests were also used to compare the PB engraftment between *MxCre/Meis1*^*fl/+*^ and *MxCre/Meis1*^*fl/fl*^ mice prior to and following PolyI:C induction. LTRC, CRU and LTC-IC frequency and testing for significant differences between groups was performed using the Extreme Limiting Dilution Analysis (ELDA) online tool (http://bioinf.wehi.edu.au/software/elda), which utilizes Poisson distribution analysis [16]. As described above, for the Affymetrix expression analysis, data were first normalized and summarized using the Affymetrix Expression Console software then adjusted using ComBat [14] to remove batch effects attributable to litter-to-litter variability rather than *Meis1* status. Samples were compared by two-tailed T-tests with unequal variance followed by Benjamini-Hochberg correction for multiple hypothesis testing [15].

## Results

### Validation of conditional deletion of Meis1

**Fig 1:**
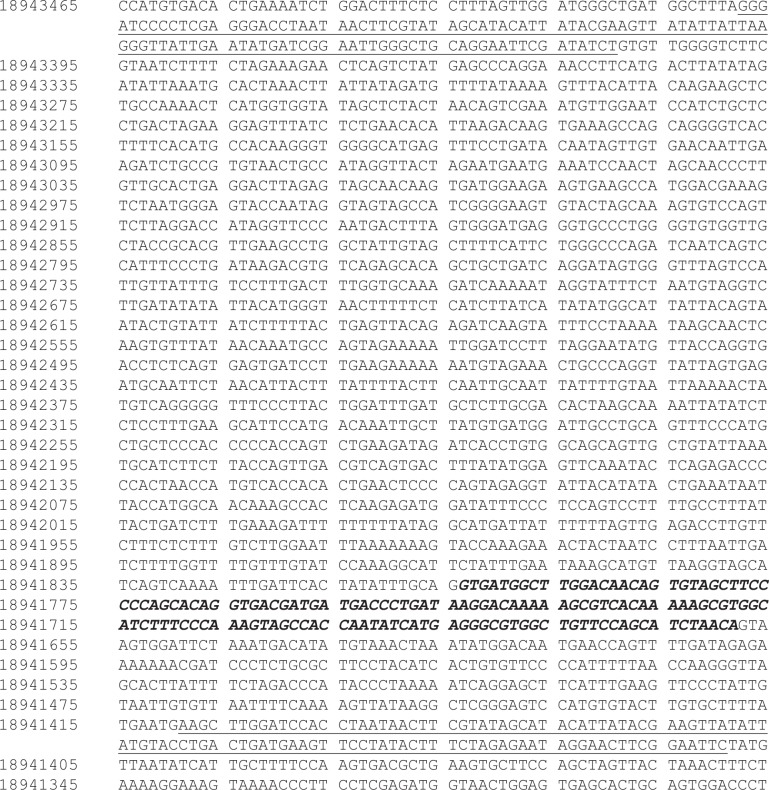
Targeted *Meis1*^*fl/fl*^ locus. The sequence is numbered according to the wild-type sequence on the antisense strand of chromosome 11 according to the Dec. 2011 (GRCm38/mm10) sequence from the Mouse Genome Reference Consortium. Non-wild-type sequence introduced by the targeting vector is underlined while the loxP sites are highlighted in grey. Exon 8 is denoted in italicized-bold type.

Male *B6-Meis1*^*tgloxP/+*^ (*Meis1*^*fl/+*^) mice were a generous gift from Drs. N. Jenkins and N. Copeland. Given limited annotation of the creation of these mice at the time of receipt, we carried out detailed molecular characterization to map the floxed *Meis1* locus (Fig 1) and validated probes for Southern blotting and primers for Q-PCR and RT-PCR to monitor allelic collapse between the loxP sites at both the DNA and RNA level (Table C in S1 File). *Meis1*^*fl/+*^ mice were then bred onto two inducible Cre recombinase strains, B6;129*Gt(ROSA)26Sor*^*tm1(cre/ERT)Nat*^/J and the B6.Cg-Tg(Mx1-cre)1Cgn/J to generate 4-OHT responsive B6-*Meis1*^*fl/fl*^*/* B6;129*Gt(ROSA)26Sor*^*tm1(cre/ERT)Nat*^/J (*ERTCre/Meis1*^*fl/fl*^) and PolyI:C responsive B6-*Meis1*^*fl/fl*^/ B6.Cg-Tg(Mx1-cre)1Cgn/J mice (*MxCre/Meis1*^*fl/fl*^). Mice from both strains were born in expected numbers and appropriate frequencies, suggesting that there was no selection against untreated *MxCre/Meis1*^*fl/fl*^ or *ERTCre/Meis1*^*fl/fl*^ germ cells or embryos *in vivo*.

A variety of induction schemes for both the *MxCre* and *ERTCre* models have been reported in the literature [17–21]. Starting with the *MxCre/Meis1* model, we first tested three 300μg PolyI:C injections spaced 48 hours apart and measured *Meis1*^*fl/fl*^ deletion by Southern blot analysis. In these mice the deletion was variable with as little as 46% deletion in spleen and 86% in BM (Fig A in S1 File). An increase in dosing to 9 injections at the same 48 hour interval resulted in >98% deletion of the floxed *Meis1* allele in BM as measured by Southern blot (Fig A in S1 File). Based on these results, an induction regimen for *MxCre/Meis1* of 9 injections of 300μg PolyI:C every 48 hours was selected for future experiments.

The induction scheme for *ERTCre/Meis1* (1mg 4-OHT/mouse every 48 hours for 6 injections) was initially chosen based on published reports [19] and local experience. This scheme also yielded >98% deletion of the floxed *Meis1* allele in BM as measured by Southern blot (Fig A in S1 File) and >500-fold reduction in exon 8-containing mRNA (data not shown). Using primers within exon 7 and 11, a truncated Meis1 mRNA containing the predicted frame-shift mutation and premature stop codon in exon 9 was verified in treated *ERTCre/Meis1*^-/-^ and *ERTCre/Meis1*^-/+^ mice (Fig B in S1 File).

### Meis family member expression in sorted wild-type mouse BM populations

To gain a better appreciation of hematopoietic cell types most likely to be affected by Meis1 deletion, a series of experiments were carried out to assess the level of Meis1 expression by Q-RT-PCR in highly purified sub-populations spanning very primitive, HSC-enriched to late lineage-positive cells (Table B in S1 File for purification details). These analyses were also extended to Meis family members including Meis2, Meis3, Prep1 and Prep2.

**Fig 2:**
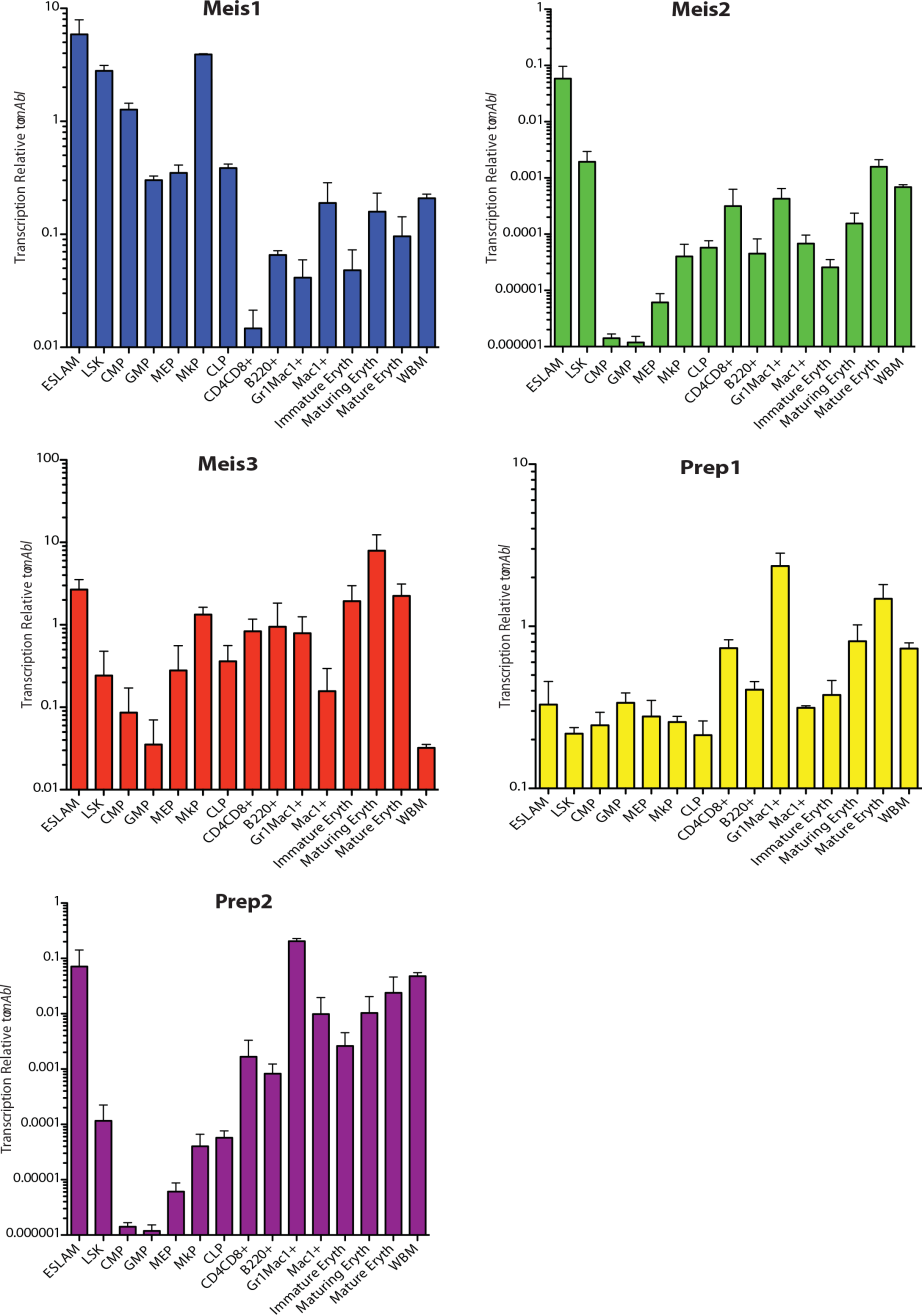
MEIS family expression in purified hematopoietic subsets. Populations enriched for specific hematopoietic subsets were sorted by FACS. MEIS family expression was interrogated in these subsets using Q-RT-PCR following a gene-specific pre-amplification. n = 3 for each of the cell lineages studied.

As shown in Fig 2, *Meis1* was detected at the highest levels in purified HSC populations (EPCR^+^CD48^-^CD45^+^CD150^+^ (ESLAM) HSC, LSK HSC) and down-regulated as cells undergo lineage commitment to common myeloid and lymphoid progenitors. High levels of *Meis1* were also present in megakaryocyte progenitors (MkP) similar to that in HSCs. *Meis1* was minimally expressed in the mature cell populations examined. With the exception of up-regulation in MkP, the patterns of expression for Meis2, Meis3 and Prep2 were generally similar to Meis1 but with expression levels dramatically lower consistent with a predominant role for Meis1 in hematopoiesis notably in HSC/early progenitors and potentially in early megakaryocytic development. *Prep1* was detected at the highest levels in granulocyte progenitors (Gr1^+^Mac1^+^) and escalating levels in subsequent steps of erythroid maturation suggestive of potential role in later stages of hematopoiesis.

### Loss of Meis1 in adult mice perturbs peripheral blood composition in both *MxCre/Meis1*^*-/-*^ and *ERTCre/ Meis1*^*-/-*^ models

**Fig 3:**
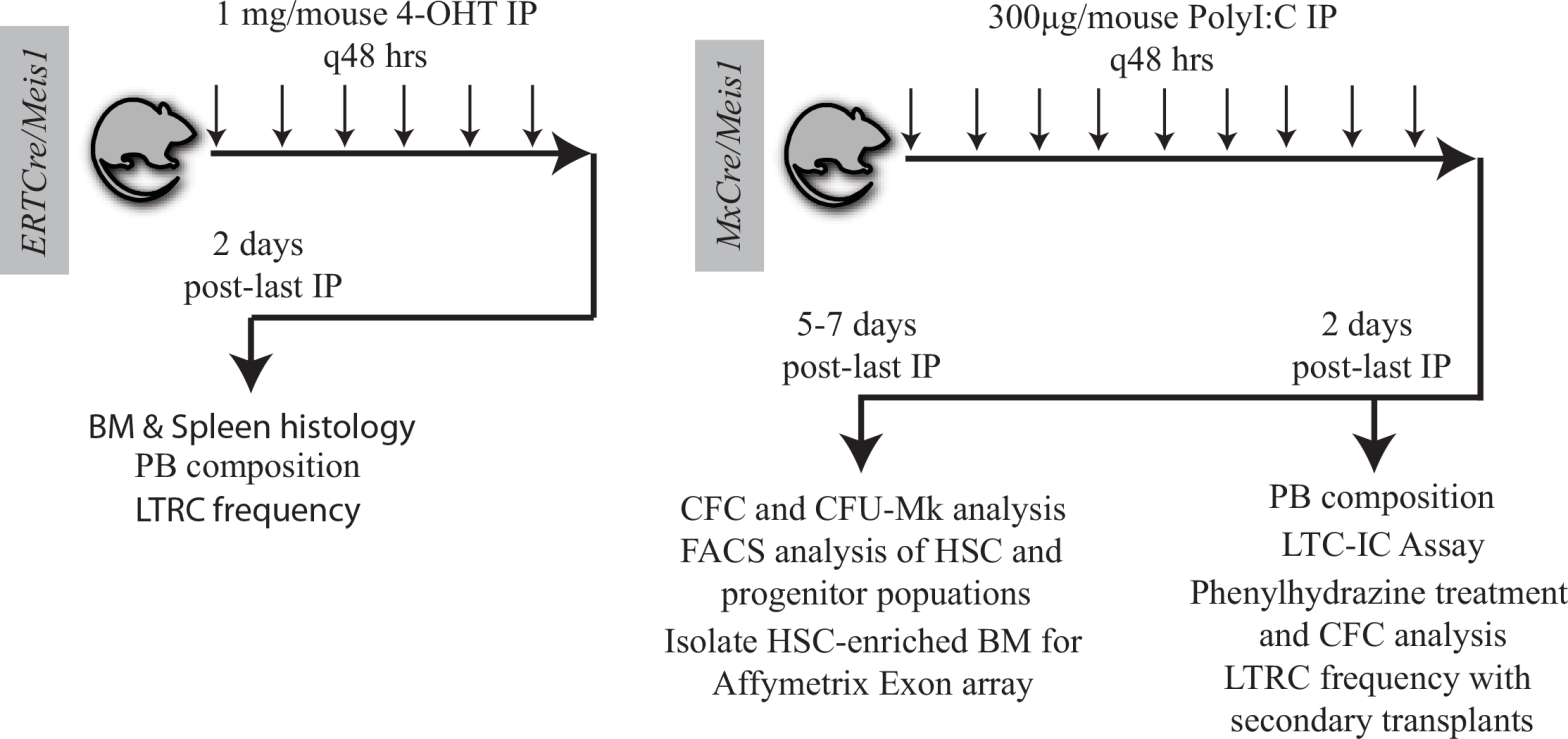
Cre induction schemes and summary of experiments performed using *ERTCre/Meis1*^*fl/fl*^ and *MxCre/Meis1*^*fl/fl*^ mice.

A first series of experiments focused on identifying effects early after *Meis1* deletion (Fig 3). *ERTCre/Meis1* mice were bled two days after the last of six 4-OHT injections. At this time, 3 of 13 *ERTCre/Meis1*^*-/-*^ mice were found to be moribund and euthanized along with litter matched Cre-expressing control mice. Data from *ERTCre/Meis1*^*-/+*^ and *ERTCre/Meis1*^*+/+*^ mice are clustered in this analysis as no significant difference was found between the groups and both had significant differences compared to *ERTCre/Meis1*^*-/-*^ mice when compared individually.

**Fig 4:**
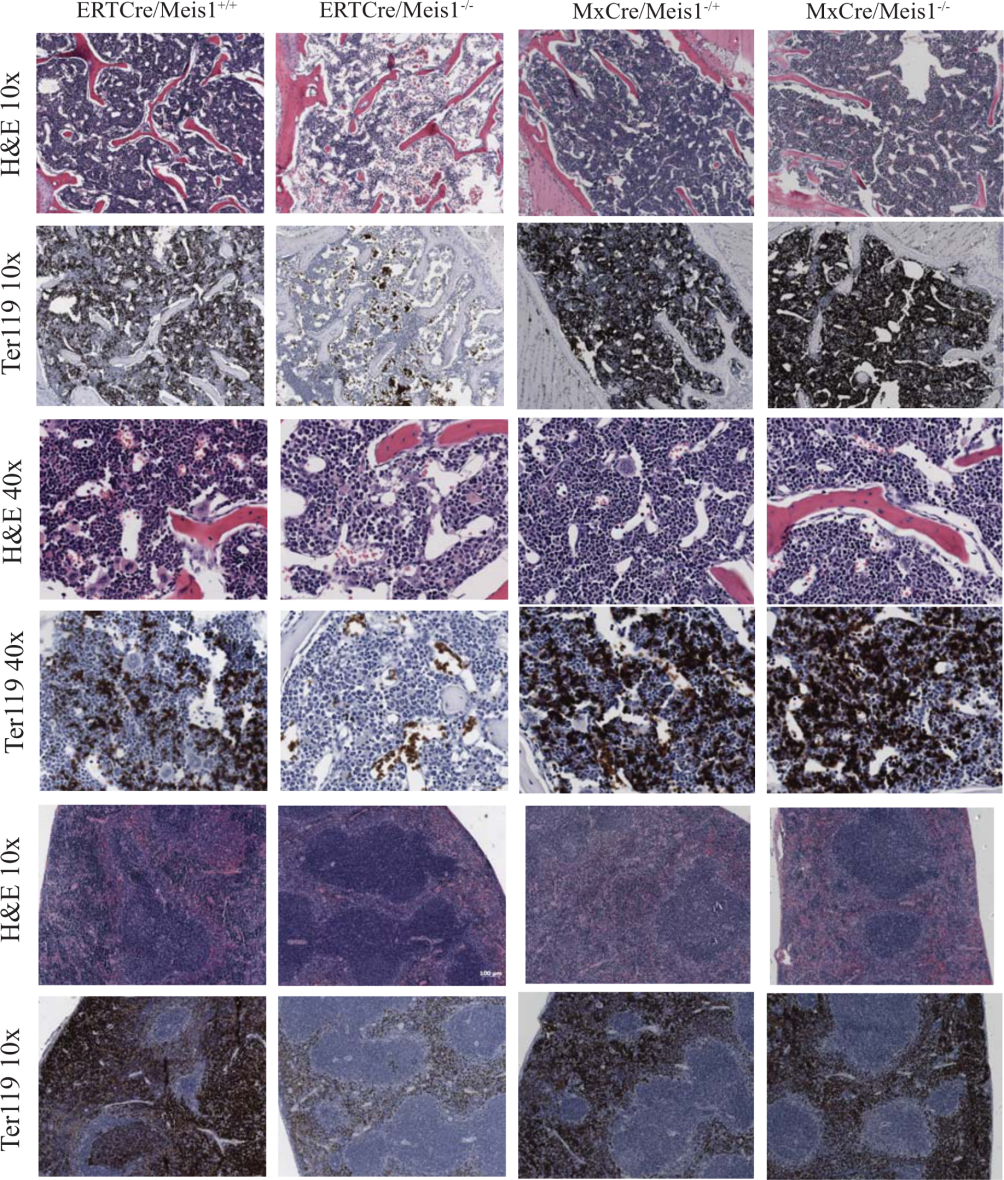
Loss of *Meis1* results in histological changes to the PB and BM composition of *MxCre/Meis1* mice. BM and spleen cross-sections of moribund *ERTCre/Meis1*^*-/-*^ and *MxCre/Meis1*^*-/-*^ mice stained with hematoxylin & eosin (H&E) and anti-Ter119 antibody, compared to *ERTCre/Meis*^*-/+*^ and *MxCre/Meis*^*-/+*^ controls.

**Fig 5:**
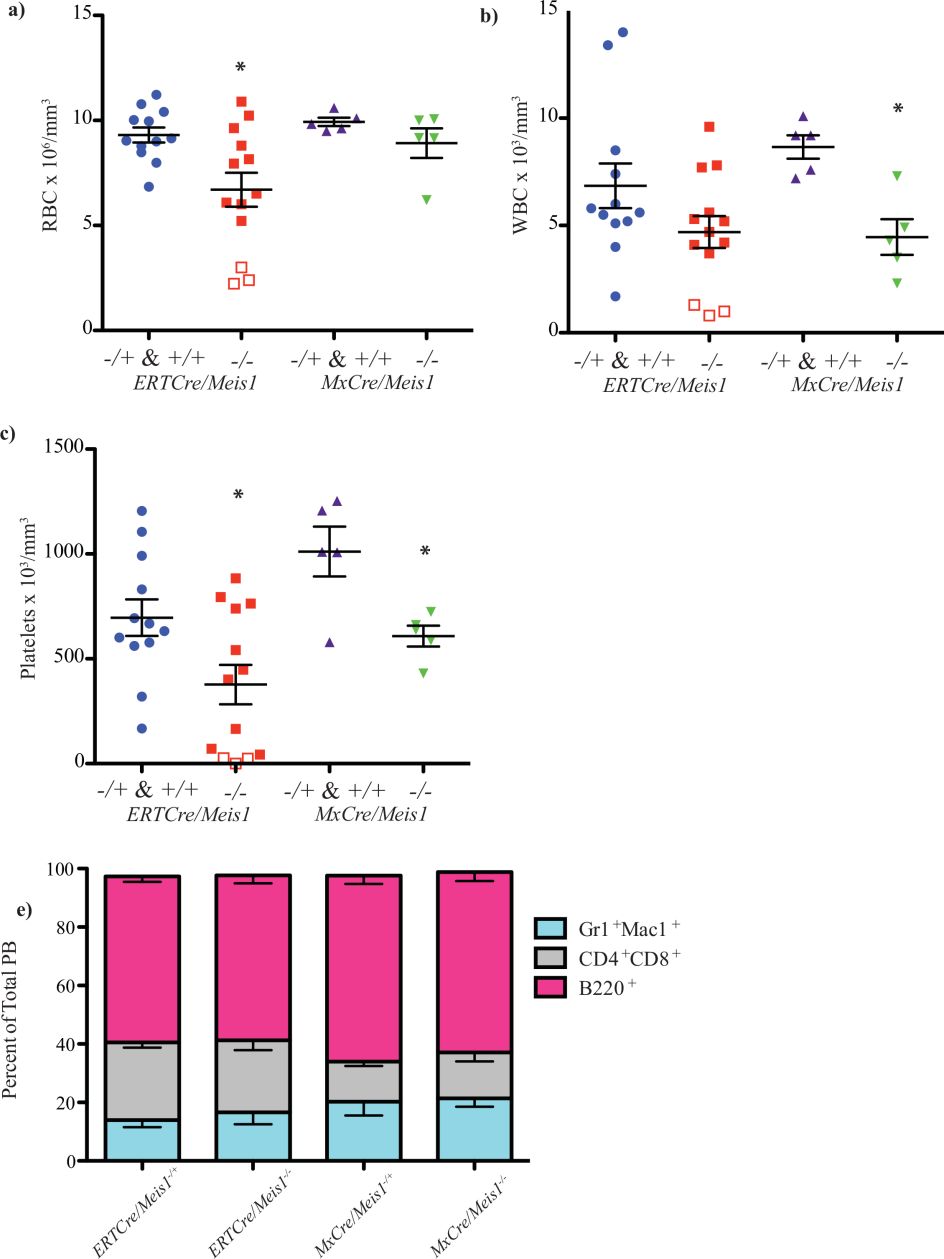
Loss of *Meis1* results in changes to the PB and BM composition of *ERTCre/Meis1* and *MxCre/Meis1* mice. **a)** Loss of *Meis1 in vivo* results in a significant decrease in red blood cells in the PB in *Meis1* ^*-/-*^ mice. *ERTCre/Meis1* ^*-/-*^ (n = 13) compared to *ERTCre/Meis1*^-/+ & +/+^ (*p =* 0.01; *ERTCre/Meis1*^*+/+*^ n = 3; *ERTCre/Meis1*^*-/fl*^ n = 10). The three *ERTCre/Meis1*^*-/-*^ mice with the lowest RBC counts were euthanized due to pallor and lethargy and had profound reductions in BM cellularity, that is, less than 1.3x10^6^ nucleated cells per trunk (2x femur, 2x iliac crest, 2x tibia). Moribund mice are represented as hollow squares on the graph. *MxCre/Meis1*^*-/-*^ mice (n = 5) show no such reduction when compared to *MxCre/Meis1*^*+/+*^(n = 2) and *MxCre/Meis1*^*-/+*^ (n = 3) mice. **b)** Loss of *Meis1* results in a reduction of white blood cells (WBC) in *MxCre/Meis1* ^*-/-*^ mice compared to *MxCre/Meis1* ^-/+ & +/+^ (*p =* 0.004, *n* = 5) 2 days after the final PolyI:C injection. **c)** Loss of *Meis1* results in a reduction of peripheral platelets (PLT) in *MxCre/Meis1* ^*-/-*^ *ERTCre*^*+*^*/Meis1* ^*-/-*^ and mice compared to *MxCre*^*+*^ and *ERTCre*^*+*^ control mice 2 days after the final PolyI:C/4-OHT injection (*p =* 0.02, and *p* = 0.02, respectively). **d)** Lineage distribution in the PB of treated mice, 2 days following the last injection. No significant differences were found.

BM from *ERTCre/Meis1*^*-/-*^ moribund mice (n = 3) showed a marked reduction in cellularity, with less than 1.3x10^6^ nucleated cells per trunk (2 femurs, 2 tibias and 2 iliac crests) compared to 1.9x10^7^ nucleated cells in controls (*p<*0.04), fatty replacement of BM (Fig 4, Fig 5B) and a marked reduction in nucleated erythroid progenitors as demonstrated by Ter119 staining on fixed tissues (Fig 4). Although there was no significant reduction in BM nucleated cell counts for the remaining 10 surviving *ERTCre/Meis1*^*-/-*^ mice (Fig 5B), there was a significant reduction in the average number RBCs in the PB of *ERTCre/Meis1*^*-/-*^ mice, even when moribund mice were excluded from the analysis (Fig 5A). The distribution of mature nucleated cell types remained largely unchanged (Fig 5D). Overall, there was also a significant reduction in the number of platelets in the PB of *ERTCre/Meis1*^*-/-*^ mice compared to controls (Fig 5C). When analyzed by gender, for this and subsequent analyses reported in the text, no significant difference was found between male and female mice in either the *ERTCre/Meis1* or *MxCre/Meis1* mice. Together these results reveal that deletion of *Meis1* in an adult mouse results in a rapid and marked decrease in RBC and platelet numbers and in at least some mice an extreme situation of BM hypoplasia, notably in early erythroid and megakaryocytic cells.

A similar early time course study was also carried out using the *MxCre/Meis1*^*fl/fl*^ model. In contrast to the *ERTCre/Meis1*^*fl/fl*^ model, no early morbidity was detected for *MxCre/Meis1*^*-/-*^ mice; and as well, marrow and spleen cellularity and histology remained unchanged compared to controls (Fig 4) as assessed 2 days after the end of Cre recombinase induction. There were however significant reductions in WBC and platelet numbers (Fig 5B and 5C). Thus in both the *MxCre/Meis1*^*-/-*^ and *ERTCre/Meis1*^*-/-*^ mouse models, loss of *Meis1* was associated with a rapid decrease in late erythroid and/or megakaryocytic/platelet numbers strongly suggestive of a role for *Meis1* at the late progenitor stage of hematopoiesis in these lineages.

### Loss of Meis1 impairs generation of megakaryocyte and erythroid progenitors

**Fig 6:**
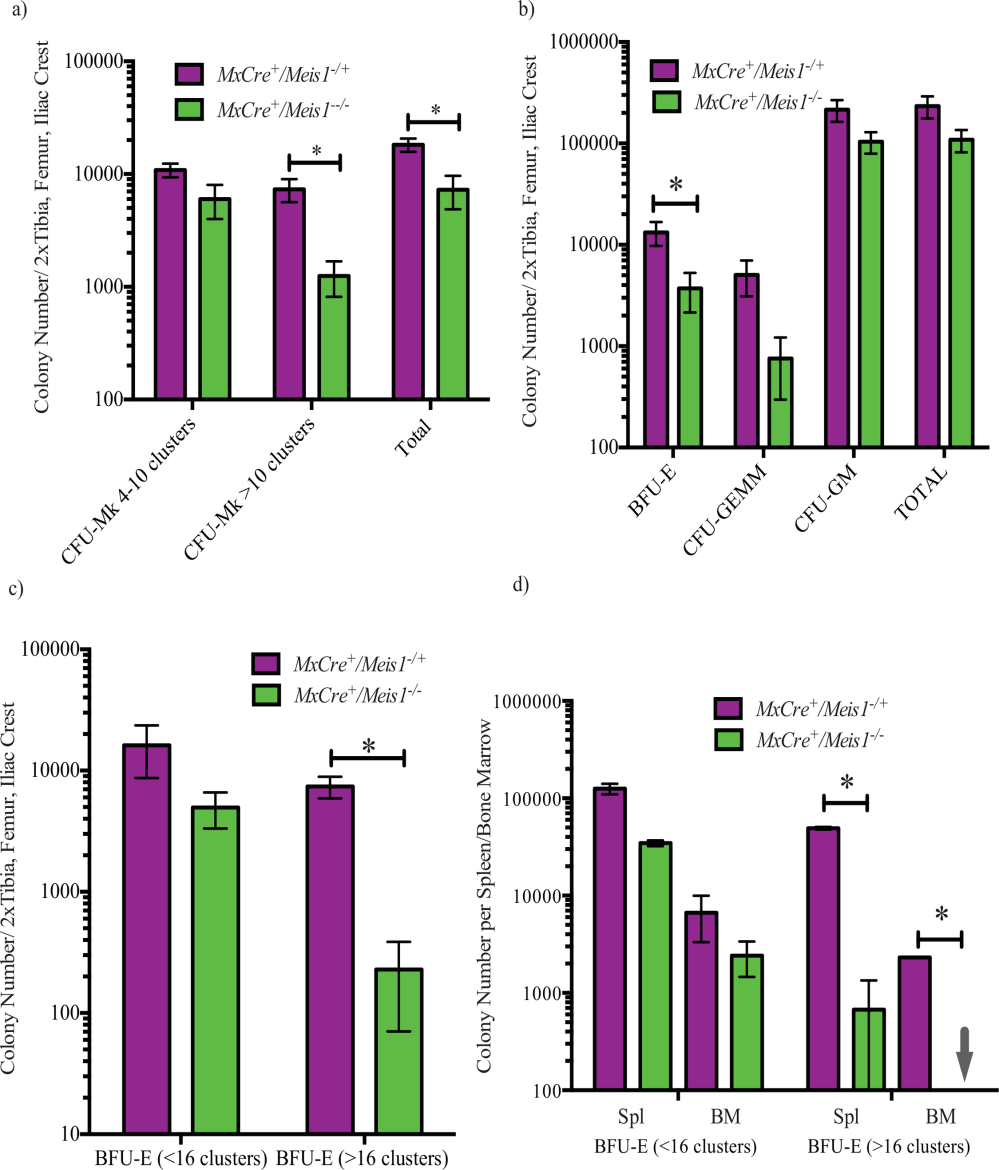
Colony forming cell (CFC) capacity is selectively reduced in the absence of *Meis1*. **a)** CFU-megakaryocytic (CFU-Mk) collagen cultures demonstrate deficiency in the ability of *MxCre/Meis1*^*-/-*^ BM to generate CFU-Mk (*p* = 0.007, *n =* 7), most notably a 9-fold reduction in large CFU-Mk composed of >10 clusters per colony (*p* = 0.01, *n* = 7). **b)** BM from *MxCre/Meis1*^*-/-*^ mice (n = 7) had a 4-fold reduced number of burst-forming erythroid (BFU-E, *p* = 0.04) and a 7-fold reduced number of CFU-granulocyte-erythrocyte-monocyte-megakaryocyte capacity (CFU-GEMM, *p* = 0.05). **c)** BFU-E impairment in *MxCre/Meis1*^*-/-*^ BM is further demonstrated in erythroid-specific methylcellulose media. *MxCre/Meis1*^*-/-*^ marrow had 32-fold fewer BFU-E with a potential of >16 clusters per colony (*p =* 0.003, *n* = 7). For both CFC and BFU-E assays, mice were euthanized and cells plated 5 days after the final PolyI:C injection. **d)** Phenylhydrazine treated *MxCre/Meis1*^*-/-*^ spleen cells have a 73-fold reduced ability to form large (>16 clusters/colony) BFU-E (*p* = 0.006, *n* = 3) compared to *MxCre/Meis1*^*-/+*^. BM showed a >2000-fold reduction in both small and large BFU-E colonies (*p* = 0.002, *n* = 3). Mice were euthanized 4 days after the phenylhydrazine and thus 6 days following the final PolyI:C injection in two replicate experiments.

To examine more closely the impact of Meis1 deletion on the megakaryocytic lineage, we assessed CFU-Mk numbers and proliferative potential from whole BM 7 days following the end of PolyI:C induction in *MxCre/Meis1* mice. Loss of *Meis1* resulted in a reduction of total CFU-Mk (*p* = 0.007, *n =* 7) compared to *MxCre/Meis1*^*-/+*^ mouse marrow. This reduction was most pronounced (6-fold) for CFU-Mk with high proliferative potential that form colonies composed of >10 cell clusters (*p* = 0.01, *n* = 7, Fig 6A). This loss of CFU-Mk is consistent with the drop in platelet number seen in the PB of *MxCre/Meis1*^*-/-*^.

To examine if this reduction in progenitor number was exclusive to the megakaryocytic lineage or extended to less differentiated myeloid parent populations and their progeny, BM CFC assays were carried out on *MxCre/Meis1*^*-/-*^ BM cells 5 days after the end of induction. Compared to *MxCre/Meis1*^*-/+*^ controls, the total colony number was not significantly reduced in *MxCre/Meis1*^*-/-*^ BM, but there was a 4-fold reduction in the number of large BFU-E derived erythroid colonies (*p* = 0.04, *n* = 7) and a 7-fold reduction in the number of multi-lineage colonies derived from CFU-GEMM (*p* = 0.05, *n* = 7) (Fig 6B). The reduction in the erythroid progenitor population was even more evident when methylcellulose media optimized to support BFU-E colony formation was used (Fig 6C). In this case, while total BFU-E colony number was unchanged compared to control, large colonies of greater than 16 cell clusters indicative of colonies derived from more primitive progenitors were reduced by 32-fold (*n* = 7, *p* = 0.003). The significant reduction in large BFU-E and CFU-Mk colonies in *MxCre/Meis1*^*-/-*^ mice suggests a role for *Meis1* in the proliferative potential of these progenitors, although it is unclear if it is at the level of a shared megakaryocyte-erythroid progenitor (MEP) or in the individual erythroid (EP) and megakaryocyte lineages (MkP).

To further investigate the erythroid defect seen in these mice, we used an *in vivo* model of hemolytic anemia induced by phenylhydrazine (PHZ) to generate a proliferative stress on erythroid progenitors. Forty-eight hours after the last of 9 PolyI:C injections, *MxCre/Meis1* mice were given an IP injection of PHZ (60mg/Kg) and euthanized 4 days later. While spleen and marrow cellularity were comparable between treated control and *MxCre/Meis1*^*-/-*^ mice (data not shown), erythroid colony numbers were greatly reduced in *Meis1*^*-/-*^ mice following PHZ treatment (Fig 6D). Again, this was particularly evident for large erythroid colonies for which there was a 73-fold reduction in number from spleens of *MxCre/Meis1*^*-/-*^ mice compared to *MxCre/Meis1*^*-/+*^ (p = 0.006, n = 2). Moreover, there were no colonies with greater than 16 clusters from the marrow of *Meis1* deficient mice, implying a greater than 2,000-fold loss (*p* = 0.002, *n* = 2) (Fig 6D). The BFU-E proliferation defect under normal and stress conditions demonstrate that *Meis1* is required for efficient erythropoiesis.

To examine the possibility that erythroid progenitors, quantified by *in vitro* CFC assay from *MxCre/Meis1*^*-/-*^ mice were derived from progenitors that escaped Cre-mediated *Meis1* deletion, individual BFU-E, CFU-GM and CFU-GEMM derived colonies were plucked and examined for *Meis1* deletion by PCR. All 40 colonies examined were collapsed around exon 8. This suggests that although the expansion of BFU-E and multi-potential progenitors is impaired in the absence of *Meis1*, subsequent downstream proliferation and differentiation are not overtly impaired. Together these data point to a major if not absolute requirement for *Meis1* in the window of early erythroid and megakaryocytic progenitor (and possibly at the MEP stage) down to the intermediate stage of committed erythroid and late megakaryocytic development.

### *Meis1*^*-/-*^ results in a loss of hematopoietic stem cells and common myeloid progenitors

**Fig 7:**
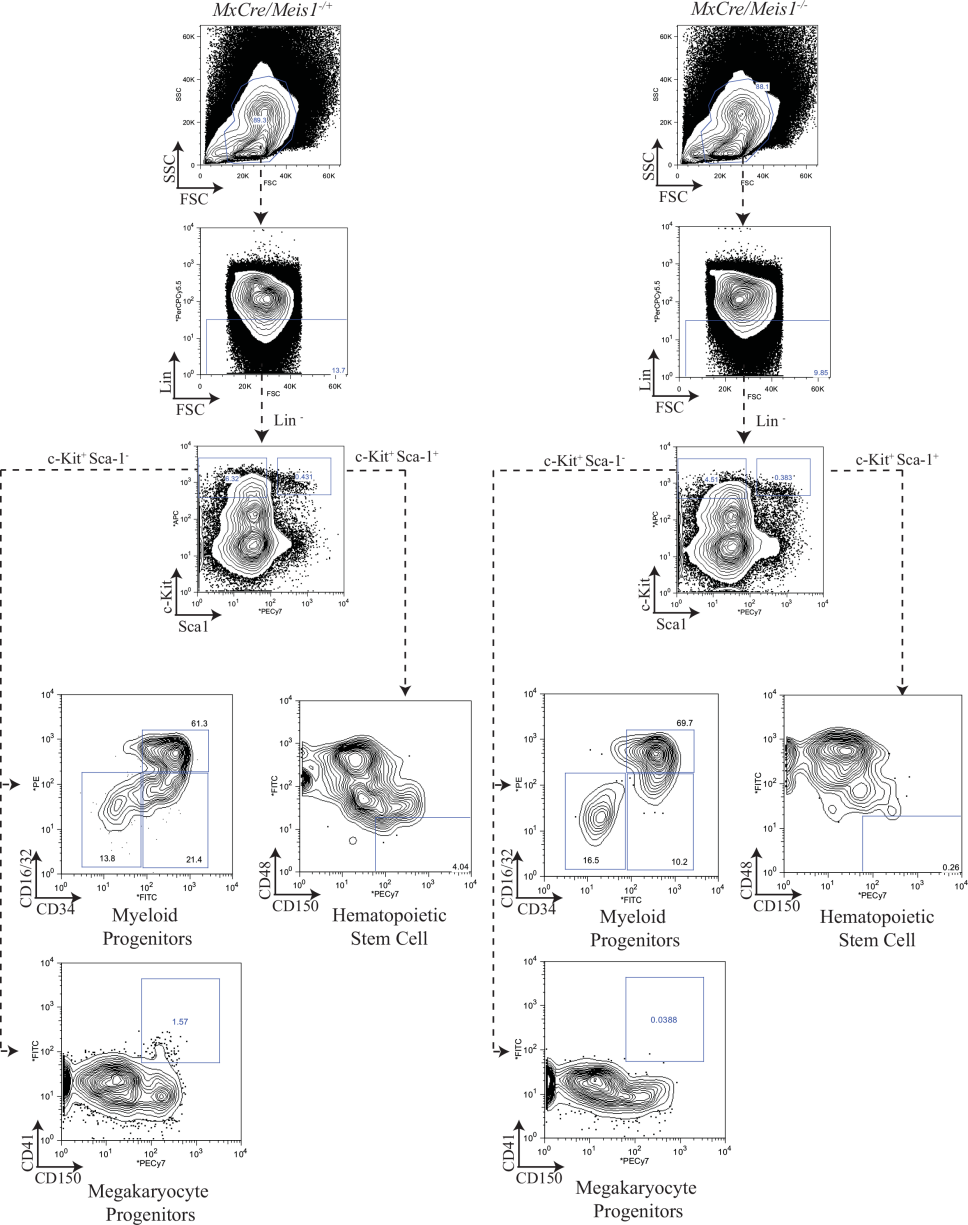
Gating strategy and representative plots for myeloid progenitors, HSCs and MkP from *MxCre/Meis1*^*-/-*^ and *MxCre/Meis1*^*-/+*^ BM

**Fig 8:**
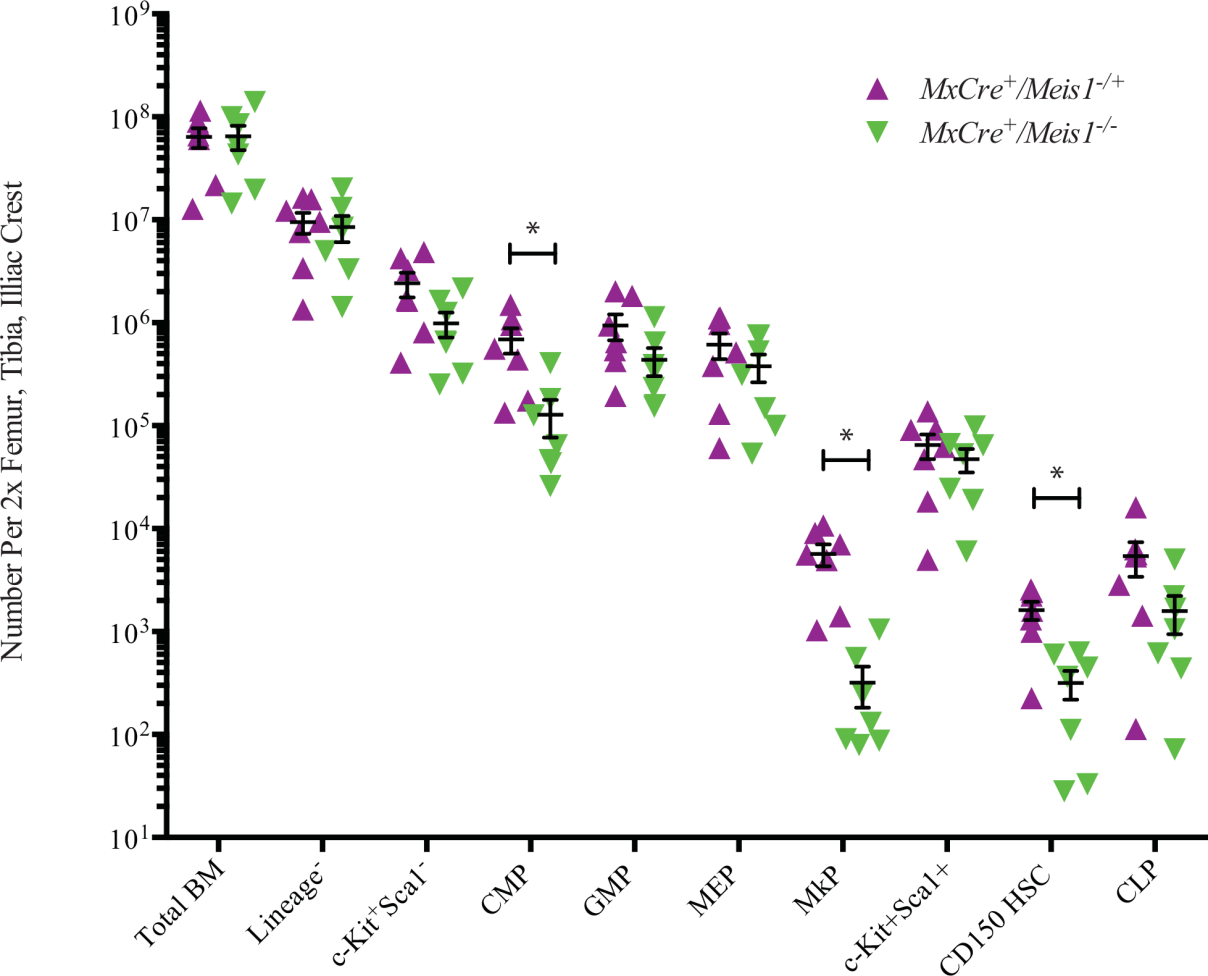
Loss of *Meis1* results in phenotypic changes in mouse BM. Absolute number of phenotypically defined populations in the BM of *MxCre/Meis1*^*-/-*^ and *MxCre/Meis1*^*-/+*^ mice. There was a 9-fold reduction in CMP (*p* = 0.04, *n* = 7), 11-fold reduction in MkP (*p =* 0.02, *n* = 7) and 5-fold reduction in HSC (*p =* 0.005, *n* = 7) enriched populations.

As an initial approach to examine the immediate impact of *Meis1* deletion on a broader spectrum of hematopoietic cells including the most primitive HSC populations, BM cells were subjected to detailed immunophenotype analyses 5 to 7 days after induction in *MxCre/Meis1*^*-/-*^ mice and *MxCre/Meis1*^*-/+*^ controls. Immunophenotyping was carried out as previously employed to isolate subpopulations for Meis expression profiling with the addition of the LSKCD150^+^CD48^-^ immunophenotype to identify primitive HSCs at an estimated purity of 1 in 2 to 1 in 5. These analyses carried out in 5 experiments with a total of 7 mice per group, revealed significant decreases in absolute numbers per mouse of several of the progenitor populations (representative plots Fig 7; results Fig 8), despite normal numbers of nucleated cells and differentiated lineage distribution in the marrow. Consistent with our earlier results from progenitor assays, there was an 11-fold decrease in the number of phenotypically defined megakaryocytic progenitors (MkP) in the marrow of *MxCre/Meis1*^*-/-*^ mice (*p =* 0.02, *n* = 7). This reduction in progenitors was also seen for the most primitive common myeloid progenitor (CMP) compartment, with a 9-fold reduction (*p* = 0.04, *n* = 7) consistent with the decrease observed in number of BFU-E and CFU-GEMM in CFC assays. No decrease was observed in the GMP subpopulation. Together these data point to a requirement for *Meis1* in the CMP and MkP compartments but limited or no requirement at the stage of GMP and later. While there was no significant difference in the number of LSK cells (HSC frequency of ~1/50), there was a marked reduction in the more highly purified HSC subpopulation (LSKCD150^+^CD48^-^) (5-fold reduction (*p =* 0.005, *n* = 7) in *MxCre/Meis1*^*-/-*^ mice compared to *MxCre/Meis1*^*-/+*^ mice.

### *Meis1* is required for the maintenance of primitive cell populations capable of long-term hematopoiesis *in vitro* and *in vivo*

To assess the impact of Meis1 deletion on functionally defined primitive hematopoietic cells, *in vitro* LTC-IC and *in vivo* competitive repopulation assays were performed. The frequency and calculated absolute number of LTC-IC as assessed by limit dilution assay was reduced by 6-fold in the BM of *MxCre/Meis1*^*-/-*^ mice compared to *MxCre/Meis1*^*-/+*^ (assays initiated 2 days post end of induction) (Table 1). Interestingly only ~50% of CFC derived from LTC-IC assays of *MxCre/Meis1*^*-/-*^ BM showed complete *Meis1* deletion. This result is consistent with a near obligate requirement for *Meis1* at the stage of LTC-IC and early downstream progeny.

**Table 1:** LTC-IC frequency in *MxCre/Meis1*^*-/-*^ BM.

4 week LTC-IC	1 in X	Upper 95% CI	Lower 95% CI
***MxCre/Meis1***^***-/+***^	2.6 x10^4^	1.9 x10^4^	3.7 x10^4^
***MxCre/Meis1***^***-/-***^	1.6 x10^5^	7.9 x10^5^	3.2 x10^5^

**Fig 9:**
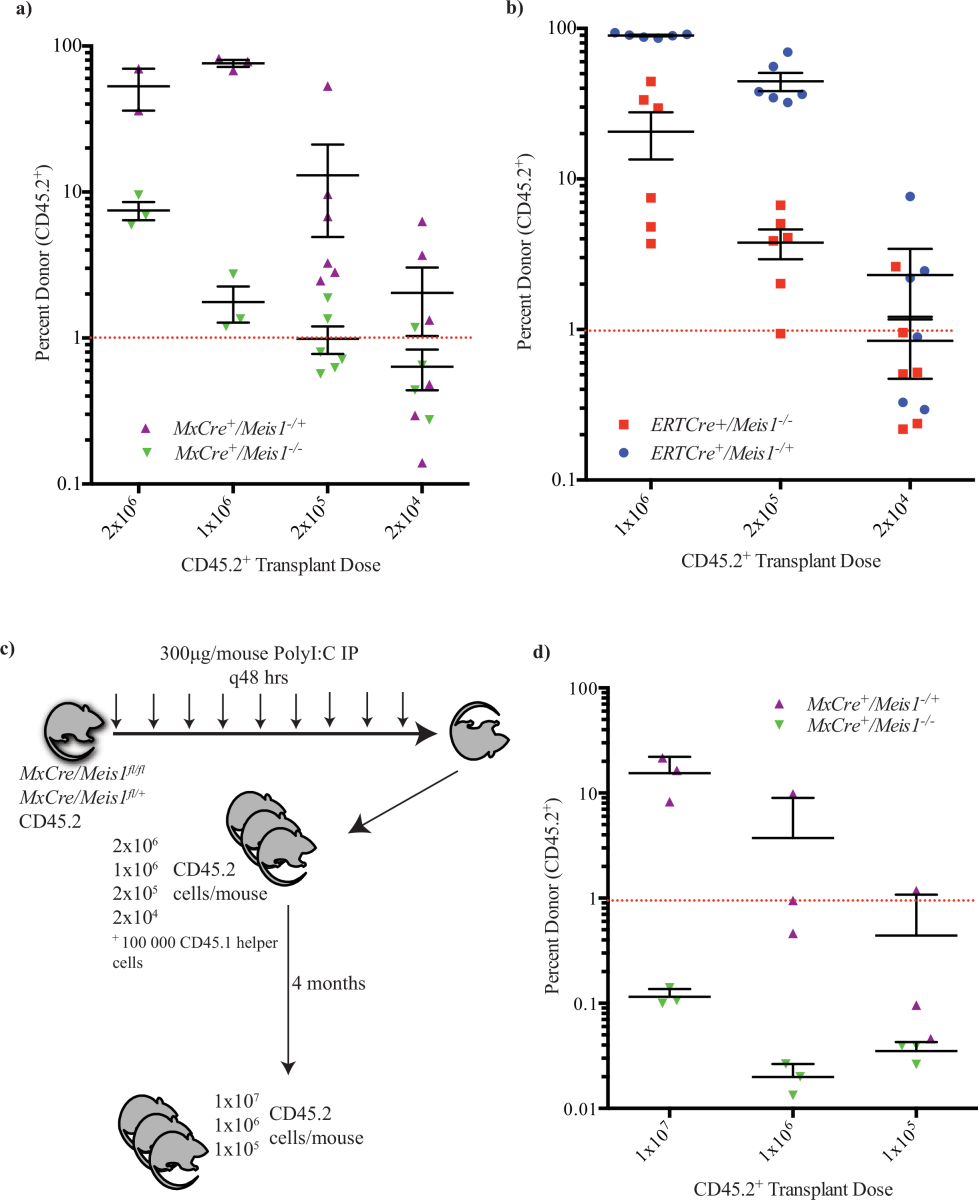
Loss of long-term repopulation and HSC self-renewal in the absence of *Meis1*. **a)** Recipient CD45.2 engraftment 16 weeks post-transplant demonstrates a 10-fold reduction in HSC capacity in *MxCre/Meis1*^*-/-*^ (n = 2 donors into 3 recipients per cell dose at 2x10^6^ and 1x10^6^, with 1 failed injection at 2x10^6^ cells. 6 recipient mice were used at 1x10^5^ and 1x10^4^ cells per mouse) BM compared to *MxCre/Meis1*^*-/+*^ (n = 2 donors into 3 recipients per cell dose at 2x10^6^ and 1x10^6^. 6 recipient mice were used at 1x10^5^ and 1x10^4^ cells per mouse; *p* = 0.009). Individual points represent individual mice from 2 independent experiments. **b)** Recipient CD45.2 engraftment 16 weeks post-transplant demonstrates a 10-fold reduction in LTRC capacity in *ERTCre/Meis1*^*-/-*^ (n = 2 donors into 6 recipients per cell dose) BM compared to *ERTCre/Meis1*^*-/+*^ (n = 2 donors into 6 recipients per cell dose; *p* = 0.00004). Individual points represent individual mice from 2 independent experiments. For both *ERTCre/Meis1* and *MxCre/Meis1* experiments, induced mice were euthanized and transplanted into donor mice 2 days following the final IP injection. **c)** Experimental outline of transplants into secondary recipients. d) Secondary recipients, 16 weeks following transplantation, demonstrate *MxCre/Meis1*^*-/-*^ HSCs are not maintained *in vivo*. *MxCre/Meis1*^*-/-*^(n = 3 pooled donors into 3 recipient mice) BM fails to engraft compared to *MxCre/Meis1*^*-/+*^ (n = 3 pooled donors into 3 recipient mice) at roughly equivalent HSC transplant doses.

To further examine the requirement for *Meis1* in the most primitive hematopoietic compartment, limiting dilution transplant assays for competitive repopulating cells were carried out. Both *MxCre/Meis1*^*-/-*^ and *MxCre/Meis1*^*+/-*^ (Fig 9A) and *ERTCre/Meis1*^*-/-*^ *ERTCre/Meis1*^*+/-*^ (Fig 9B) bulk BM cells were transplanted at different doses with competitor recipient type BM cells (100,000 cells). Donor cell contributions to the PB of recipient mice were then serially assessed at 4-week intervals to 16 weeks post-transplantation. Limit dilution analysis revealed a 10.7-fold and 19-fold reduction in the frequency of HSC in *MxCre/Meis1*^*-/-*^ (Fig 9A) and *ERTCre/Meis1*^*-/-*^ (Fig 9B) mice, respectively, when compared to *MxCre/Meis1*^*-/+*^ or *ERTCre/Meis1*^*-/+*^ mice (Table 2). Expressed in absolute numbers of HSCs per mouse, there was an average of 2900 HSCs per *MxCre/Meis1*^*-/+*^ compared to only 270 in the *MxCre/Meis1*^*-/-*^ mouse. The reduction is similar in the *ERTCre* model (5000 HSCs in *ERTCre/Meis1*^*-/+*^ mice compared to 230 in *ERTCre/Meis1*^*-/-*^ mice).

**Table 2:** Reduction in HSCs in *MxCre/Meis1*^*-/-*^ and *ERTCre/Meis1*^*-/-*^ mice.

	Mouse	HSC Frequency	Upper 95% CI	Lower 95% CI
**Primary**	*MxCre/Meis1*^*+/-*^	1 / 2.8 x10^4^	1 / 9.5 x10^3^	1 / 8.6 x10^4^
**(*p* = 0.009)**	*MxCre/Meis1*^*-/-*^	1 / 3.1 x10^5^	1 / 1.2 x10^5^	1 / 7.7 x10^5^
**Primary**	*ERTCre/Meis1*^*+/-*^	1 / 1.8 x10^4^	1 / 6.5 x10^3^	1 / 5.1 x10^4^
**(*p* = 0.00004)**	*ERTCre/Meis1*^*-/-*^	1 / 3.5 x10^5^	1 / 1.5 x10^5^	1 / 7.9 x10^5^

No differences were seen in the lineage distribution of donor-derived PB cells between *MxCre/Meis1*^*-/-*^ and *MxCre/Meis1*^*-/+*^ mice. At the time of transplantation, donor cells were confirmed to be >95% deleted for *Meis1* and Q-PCR analysis, normalized for the *Meis1*^*fl*^ allele, showed persistence of *Meis1*^*-/-*^ cells in the donor compartment at >80% (data not shown) at 16-weeks. Thus while *Meis1* loss results in a rapid and marked reduction in HSC numbers, the presence of *Meis1* does not appear to be an absolute requirement for at least limited repopulation capacity of downstream progenitors.

To examine more closely the importance of Meis1 for HSC function, secondary transplants were performed to assess HSC self-renewal and expansion in the absence of *Meis1* in the *MxCre*/*Meis1* model. In this experiment, 3 *MxCre/Meis1*^*fl/fl*^ and 3 *McCre/Meis1*^*fl/+*^ mice were treated with PolyI:C as per the established induction scheme (Fig 9C). Limiting dilution analysis in irradiated primary recipients was then performed at 2x10^6^, 1x10^6^, 2x10^5^ and 2x10^4^ cells per replicate mouse with 100, 000 helper cells. Following 4 months *in vivo*, a cohort of 3 mice transplanted with 2x10^6^ cells were sacrificed, their BM pooled and transplanted at multiple donor derived (CD45.2) doses without helper cells into irradiated secondary recipient mice (Fig 9D). The highest transplant dose of 1x10^7^ cells was expected to contain the progeny of roughly 5 HSCs (minimum 2 HSCs, maximum 12 HSCs) in the *MxCre/Meis1*^*-/-*^ arm, based on the limiting dilution results in primary recipients. Even at this dose, however, there was no detectable long-term repopulation in secondary recipients in the absence of *Meis1*. *MxCre/Meis1*^*-/+*^ cells were, however, capable of minimum maintenance of the transplanted stem cell pool. This represents, at minimum, a 25-fold reduction in the ability of HSCs lacking *Meis1* to contribute to long-term repopulation. This deficit was evident as early as 4-weeks post-transplantation into secondary recipients, suggesting HSCs lacking *Meis1* are severely impaired in their ability generate progeny with even short-term repopulating potential.

### The requirement for Meis1 for HSC is cell intrinsic

**Fig 10:**
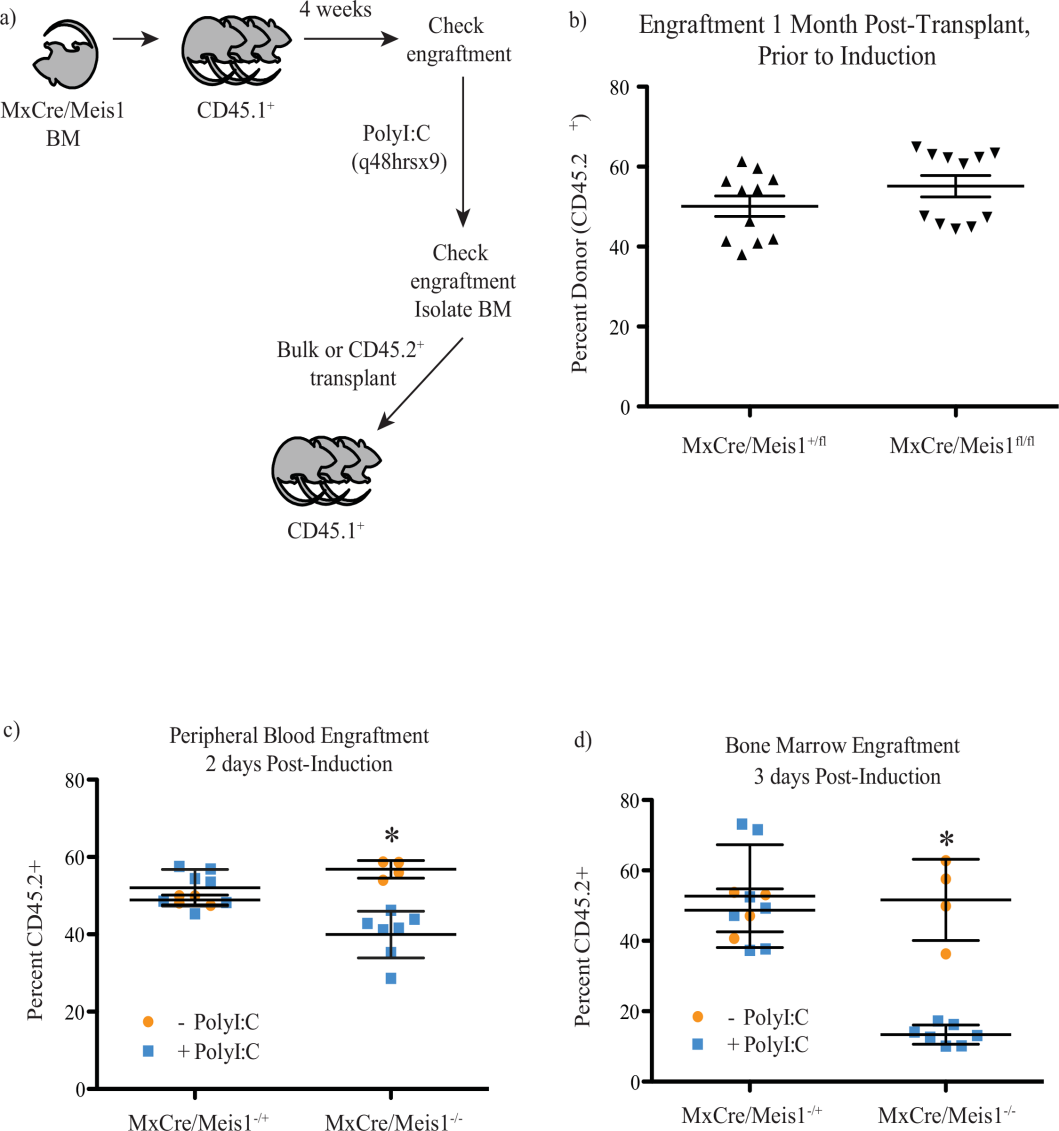
Loss of *Meis1* results in an intrinsic defect in LTRC. **a)** Experimental plan to test the cell-intrinsic requirement for *Meis1* in HSCs. **b)** PB engraftment of primary recipients 4 weeks after transplantation and prior to induction shows no difference in engraftment (n = 11). **c)** PB engraftment of primary recipients 2 days following PolyI:C administration. *MxCre*^*+*^*/Meis1*^*-/-*^ (n = 7) engraftment is reduced 17% compared to *MxCre/Meis1*^*fl/fl*^ (n = 4; *p* = 0.0005). **d)** BM engraftment in primary recipients 3 days following PolyI:C administration. *MxCre*^*+*^*/Meis1*^*-/-*^ (n = 7) engraftment is reduced 38% compared to *MxCre/Meis1*^*fl/fl*^ (n = 4; *p* = 6x10^-6^).

The decrease in HSC numbers detected following *Meis1* loss as assessed at the level of immunophenotype or functional repopulation could arise from intrinsic requirements for HSC function and maintenance and/or from cell-extrinsic requirements, for example in the stem cell niche. To examine these possibilities, we transplanted 2x10^6^ *MxCre/Meis1*^*fl/fl*^ (n = 2 donor mice into 11 recipient mice) or *MxCre/Meis1*^*+/fl*^ (n = 2 donor mice into 11 recipient mice) cells with 1x10^6^ recipient-type cells into lethally irradiated wild-type recipients 4-weeks prior to induction of Cre expression (Fig 10A). Both *MxCre/Meis1*^*fl/fl*^ and *MxCre/Meis1*^*+/fl*^ cells engrafted equivalently as read out in PB prior to Cre expression (Fig 10B). Following PolyI:C treatment, there was a 17% decrease in *MxCre/Meis1*^*-/-*^ contribution to PB 2 days after the final IP injection, whereas the levels of *MxCre/Meis1*^*-/+*^ engraftment remained unchanged compared to control mice given PBS IP (*p* = 0.0005, n = 7 PolyI:C, n = 4 PBS, Fig 10C). The decrease in engraftment by *MxCre/Meis1*^*-/-*^ was also evident in BM (38% decrease) (Fig 10D).

**Fig 11:**
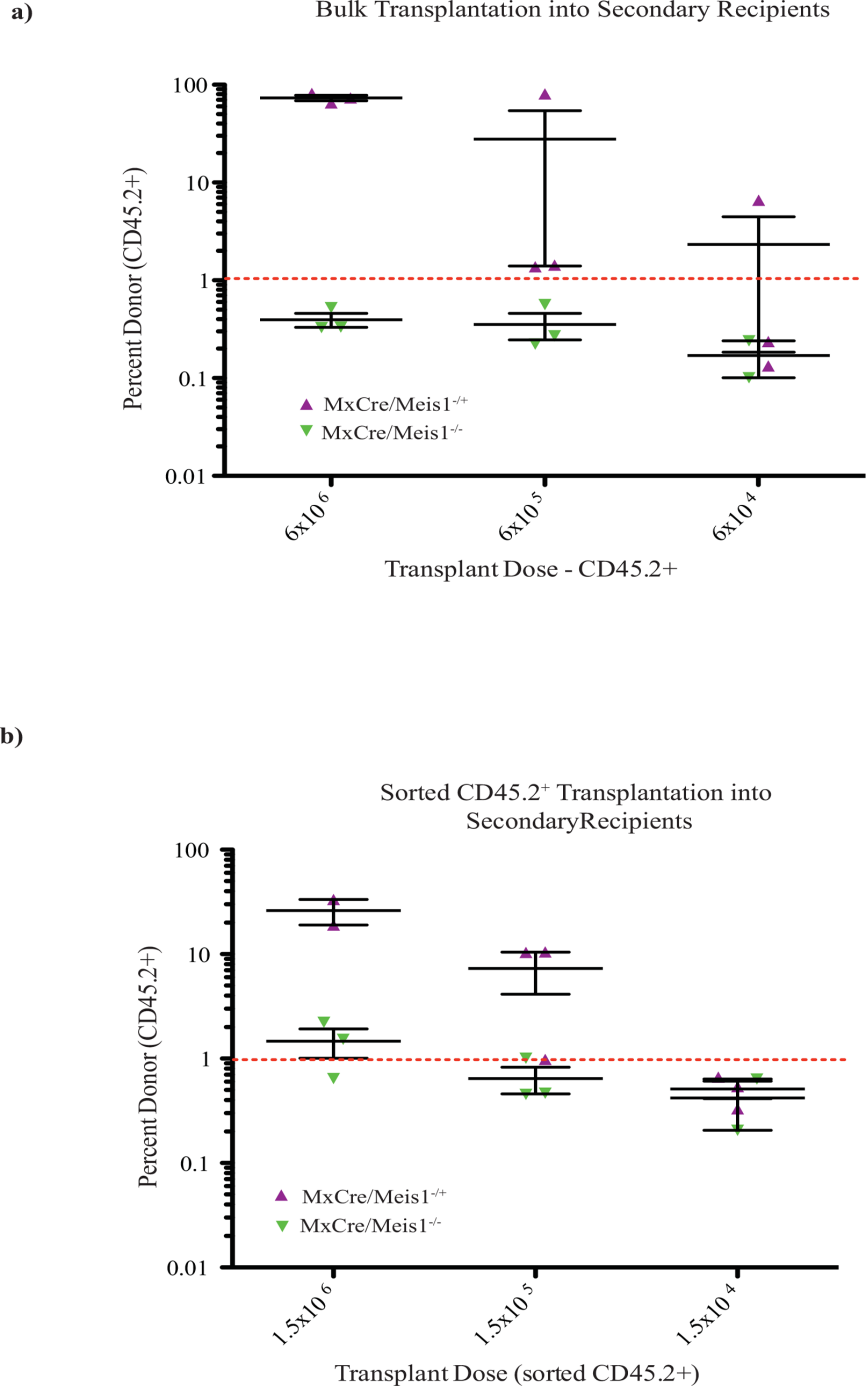
Loss of *Meis1* results in an intrinsic defect in HSC self-renewal. **a)** Engraftment of secondary recipients transplanted with bulk CD45.2 cells from primary mice with transplant dose based on the number of CD45.2 cells 24 weeks after transplantation. **b)** Engraftment of secondary recipients transplanted with sorted CD45.2 cells from primary mice 24 weeks after transplantation.

In a first experiment to determine the HSC frequency following *Meis1* deletion, the 3 recipient PolyI:C-treated mice were euthanized 3 days after the final IP injection, their BM pooled and transplanted into secondary recipients. In this initial experiment, BM cells were transplanted without separation such that there was a mixture of *MxCre/Meis1*-derived cells (CD45.2+) and recipient/competitor derived cells (CD45.1+). Cell doses were however adjusted so that equivalent numbers of *MxCre/Meis1*-derived cells were transplanted. In this setting, there was no detectable engraftment by *MxCre/Meis1*^*-/-*^ cells in the PB at 16 weeks (3 recipient mice per cell dose, Fig 11A and Table 3) in contrast to readily detected engraftment by *MxCre/Meis1*^*-/+*^ cells.

**Table 3:** HSC frequency following *in vivo* deletion of *Meis1* in primary recipients.

	CD45.2^+^	HSC Frequency	Upper 95% CI	Lower 95% CI
***MxCre/Meis1***^***+/-***^	BULK	1 / 1.4 x10^5^	1 / 3.1 x10^4^	1 / 6.2 x10^5^
***MxCre/Meis1***^***-/-***^	*p<*0.06	1 / 1.8 x10^7^	1 / 2.5 x10^6^	1 / 1.3 x10^8^
***MxCre/Meis1***^***+/-***^	SORTED	1 / 1.6 x10^5^	1 / 4.0 x10^4^	1 / 6.5 x10^5^
***MxCre/Meis1***^***-/-***^	*p*<1x10^-125^	1 / 1.0 x10^6^	1 / 2.8 x10^5^	1 / 3.6 x10^6^

By performing bulk transplants based on *MxCre/Meis1* cell number, *MxCre/Meis1*^*-/-*^ HSCs had to compete against a greater number of recipient wild-type cells than *MxCre/Meis1*^*-/+*^ cells due to the reduction in engraftment following PolyI:C treatment. The experiment was therefore repeated to compensate for increased competition by recipient cells by sorting for CD45.2^+^ BM cells prior to transplantation into secondary recipients, thereby ensuring that *MxCre/Meis1*^*-/-*^ and *MxCre/Meis1*^*-/+*^ HSCs were competing against equivalent numbers of wild-type HSC for engraftment. PolyI:C-treated primary recipient mice were euthanized, their BM pooled and CD45.2^+^ isolated by FACS transplanted into 3 secondary recipients per cell dose. The decreased frequency of HSCs in *MxCre/Meis1*^*-/-*^ cells was again apparent with a 7-fold reduction compared to *MxCre/Meis1*^*-/+*^ cells (*p*<1x10^-125^, Fig 11B, Table 3). These experiments demonstrate that the major requirement for *Meis1* in the maintenance of HSC number is likely cell intrinsic.

### Gene expression changes following loss of *Meis1* in an HSC-enriched population

To search for differentially regulated genes following Meis1 deletion, *Meis1* was deleted in *MxCre/Meis1* mice and the HSC/progenitor -enriched LSK population isolated for mRNA extraction 7 days after the final PolyI:C injection. mRNA isolated from 3 individual *MxCre/Meis1*^*-/-*^ and 3 *MxCre/Meis1*^*-/+*^ mice was amplified and subjected to transcriptome analysis using the Affymetrix Exon ST array. Following normalization for hybridization, litter batch effects and multiple hypothesis testing, 171 differentially expressed genes were identified using a 90% confidence interval (Table E in S1 File). Of these differentially expressed genes, 8 had a fold change expression greater than two-fold (Table 4).

**Table 4:** Genes with > 2-fold expression change associated with deletion of *Meis1* as determined by Affymetrix analysis.

Probe Name	Description	Adjusted t-Test	Fold change in *Meis1*^*-/-*^
**6954615**	VAMP5	0.0982	5.7767
**6805191**	OLFR4	0.0790	3.4010
**6790699**	HLF	0.0120	3.1807
**6790621**	MSI2	0.0422	2.0704
**6748889**	IL18R1	0.0790	0.4877
**6959584**	TYROBP	0.0959	0.4776
**6767782**	LILRB4	0.0959	0.2179
**6969997**	HBB-B1	0.0937	0.2050

**Fig 12:**
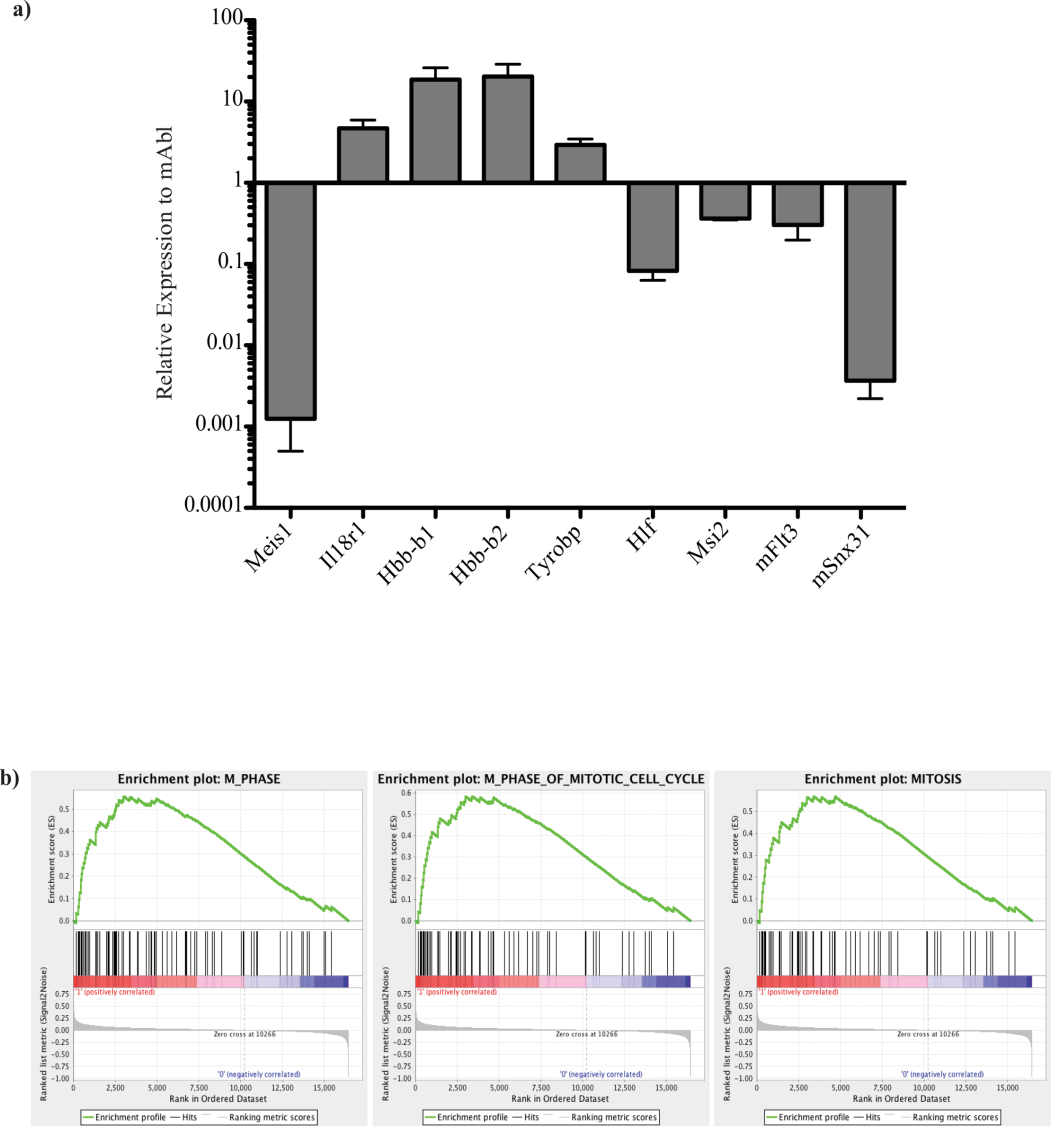
Affymetrix mouse Exon ST 1.0 analysis of gene expression changes as a result of loss of *Meis1* in an HSC-enriched population. Individual, litter-matched *MxCre/Meis1*^*fl/fl*^ and *MxCre/Meis1*^*fl/+*^ mice were induced for *Meis1* deletion with PolyI:C every 48 hours for 18 days. 7 days following the final injection, mice were euthanized, BM extracted and sorted for LSK cells by FACS. RNA was isolated from this HSC-enriched population and amplified prior to hybridization on an Affymetrix mouse Exon ST 1.0 gene expression array. Signal was normalized using Expression Console software and log2 summarization. Differentially expressed genes were identified using two-tailed t-test with multiple hypothesis correction. Changed pathways were interrogated by leading edge analysis. **a)** Validation of selected candidate target genes by Q-RT-PCR, using both sample RNA and two additional independent replicates. **b)** Leading edge gene-set enrichment analysis reveals enrichment of genes involved in cell cycle in *MxCre/Meis1*^*-/-*^ cells.

These 8 genes and others of interest selected from previous studies of *Meis1* candidate targets were validated by RT-PCR on the original samples and an additional two independent replicates (Fig 12A). Of note, 2 genes implicated in leukemia, *Hlf* and *Msi2* [22,23] are down-regulated in response to the loss of *Meis1*. Msi2 has also been reported to be up-regulated in the context of Vp16/Meis1 [24]. ChIP-Seq data for Meis1 performed in our laboratory (Eric Yung *et al*., unpublished) has also revealed MEIS1 binding sites in the body of *Msi2* and in both the body and transcription start site of *Hlf*. *Flt3* was also identified as direct target of MEIS1 in these experiments and was found to be differentially expressed by 1.5-fold between *MxCre*^*+*^*/Meis1*^*-/—*^and *MxCre*^*+*^*/Meis1*^*-/ +*^ LSK cells. *Flt3* has previously been shown to be a direct target and induced by *Meis1* overexpression and is thought to be one of several pathways by which *Meis1* influences leukemic activity [25].

Leading edge Gene Set Enrichment Analysis (GSEA; [26]) of all differentially expressed genes showed enrichment in cell cycle genes in *MxCre/Meis1*^*-/-*^ LSK cells, consistent with the loss of quiescence seen in other studies and leukemia models (Fig 12B, [6, 27]). Sets meeting a nominal *p<*0.01 and FDR of < 25% were all implicated in cell cycle M phase. Direct comparison of hypoxia-associated sets did not reach significance nor did those for megakaryopoiesis or erythropoiesis. Cell cycle analysis carried out using BrdU staining did not reveal differences in *Meis1*^*-/-*^ cells *in vitro* or *in vivo* but limited cell numbers for the critical HSC subpopulation may have compromised the sensitivity of the assay employed (data not shown).

A recent study using *Meis1* ^*-/-*^ Lin^-^ adult BM cells as input for the Affymetrix array [6] revealed down-regulation of several gene sets up-regulated in response to hypoxia, including those regulated by *Hif1α*. This is consistent with recent reports by another group [7, 28] reporting direct regulation of *Hif1α* by *Meis1* in the HSC compartment. We were unable to find evidence of *Hif1α* loss of expression by RT-Q-PCR in our sorted KSL population. We also looked in the more highly HSC-enriched LSKCD150^+^CD48^-^ population by Q-RT-PCR and found no difference in expression between *MxCre/Meis1*^*-/-*^ and *MxCre/Meis*^*-/+*^ in terms of *Hif1α* or *Hif2α* gene expression relative to *mAbl* (described later in text).

### Treatment with N-acetyl-L-cysteine rescues some of the phenotypic abnormalities seen with loss of *Meis1*

**Fig 13:**
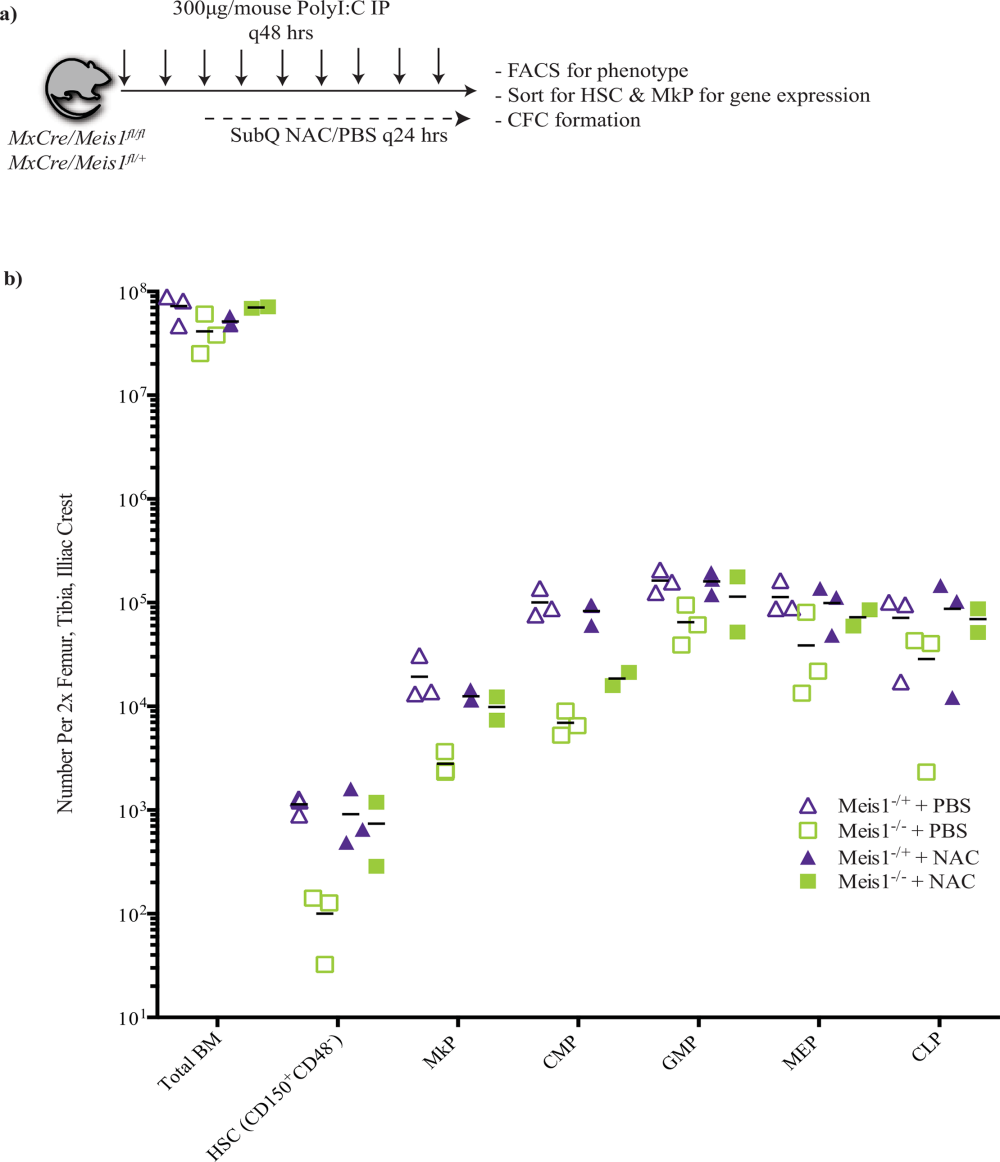
NAC treatment partially abolishes phenotypic differences between *MxCre/Meis1*^*-/-*^ and *MxCre/Meis1*^*-/+*^ mice. **a)** Experimental design. **b)** Phenotypic analysis of various cell populations by FACS between *MxCre/Meis1*^*-/-*^ and *MxCre/Meis1*^*-/+*^ mice treated with PBS and NAC. The differences between *MxCre/Meis1*^*-/-*^ and *MxCre/Meis1*^*-/+*^ mice treated with PBS are consistent with those found in earlier experiments. Phenotypic differences between *MxCre/Meis1*^*-/-*^ and *MxCre/Meis1*^*-/+*^ mice were abolished with NAC treatment with the exception of maintenance of a 4.5-fold reduction in CMP numbers.

Recent studies suggest that MEIS1 plays a role in regulation of *Hif1α* expression and resultant regulation of ROS. It is thus hypothesized that increased ROS levels and resultant damage to HSC may underlie the decrease in HSC number and function upon *Meis1* deletion [6, 7, 28]. Support for this model has been derived from studies demonstrating phenotypic rescue with N-acetyl-L-cysteine (NAC), a ROS scavenger. Despite a lack of evidence of *Hif1α* deregulation at the level of mRNA in our model, we tested whether *in vivo* administration of NAC could rescue deficits seen in the HSC, MkP and CMP populations. PolyI:C mediated Cre expression was initiated in *MxCre/Meis1*^*fl/fl*^ and *MxCre/Meis1*^*fl/+*^ mice. On the third PolyI:C injection, daily subcutaneous NAC or PBS injections were initiated (Fig 13A). Five days after the final subcutaneous injection of PBS or NAC, mice were euthanized and analyzed for level of *Meis1* deletion, phenotype, CFC capacity and gene expression in sorted HSC and MkP populations. Estimated *Meis1* deletion was comparable for control and NAC treated mice (97% versus 85% respectively). Consistent with earlier experiments, for the control PBS treated mice, HSC and MkP numbers, were reduced in *Meis1* deleted mice compared to controls (11-fold and 7-fold drop respectively; Fig 13B). CMP and GMP numbers were also reduced (14.5-fold and 2.5-fold; Fig 13B). In contrast, for NAC treated *Meis1* deleted mice there was a strong trend to normalization of phenotypically defined cell numbers compared to NAC treated *Meis1*^*-/+*^ mice, with the exception of CMPs where there remained a 4.5-fold reduction. The loss of one mouse in the NAC treated *Meis1* deleted group due to injection site irritation limited statistical analysis and would require additional experiments for confirmation. Nevertheless these preliminary findings are supportive of previous studies showing a blunting of the early effect of Meis1 deletion by NAC treatment.

**Fig 14:**
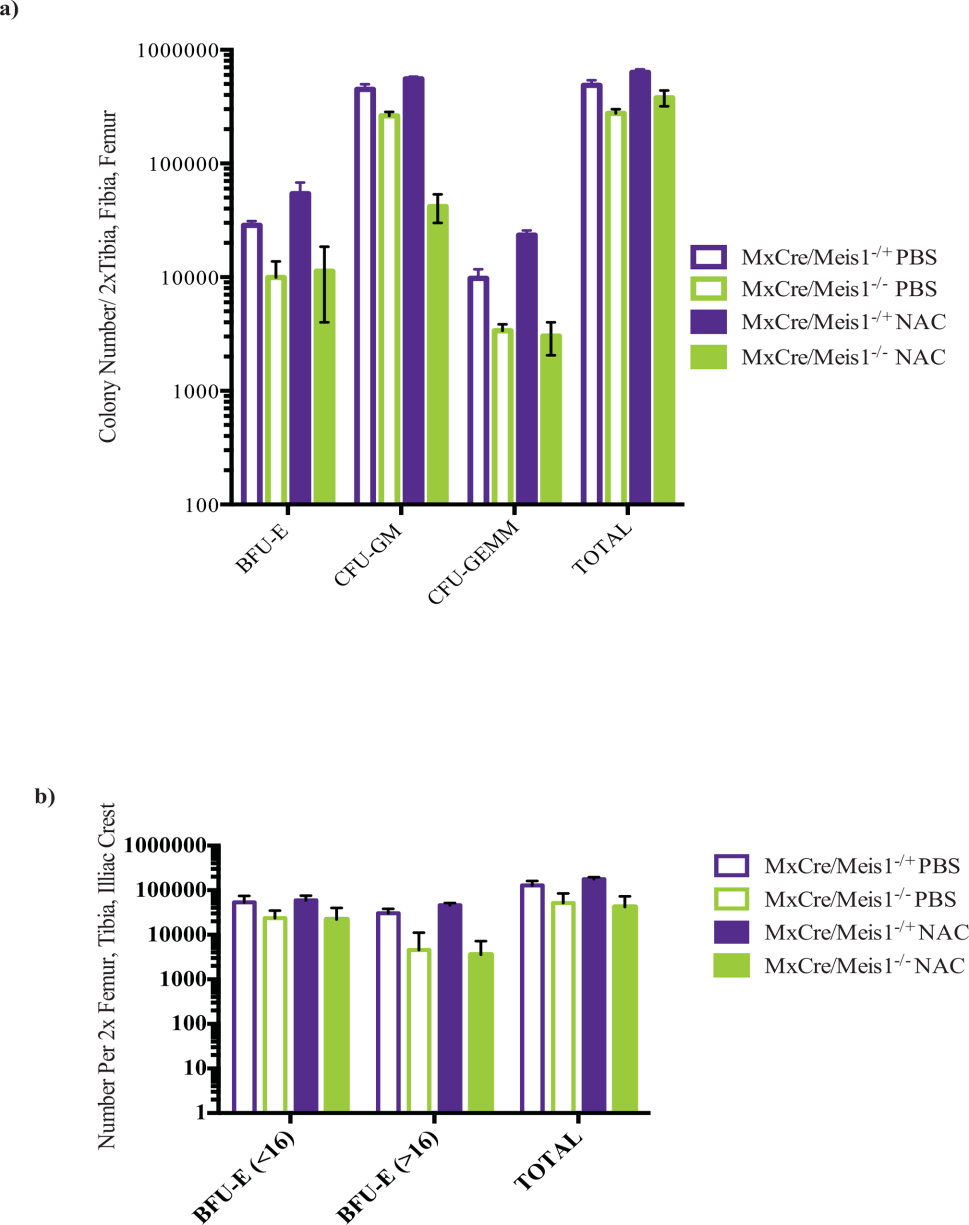
NAC treatment does not rescue committed progenitor functional differences between *MxCre/Meis1*^*-/-*^ and *MxCre/Meis1*^*-/+*^ mice. a) CFC numbers assessed in myeloid-supportive conditions remained reduced in *MxCre/Meis1*^*-/-*^ mice following *in vivo* NAC administration. b) NAC did not rescue the capacity for *MxCre/Meis1*^*-/-*^ cells to form large BFU-E (>16 clusters/colony) in erythroid supportive media (12.8-fold reduction,). Both FACS data and colony numbers are expressed as absolute numbers isolated from the trunk of mice (2 femurs, 2 tibias, 2 iliac crests).

In contrast to the observed effect of NAC treatment on certain subsets of phenotypically defined hematopoietic cells, NAC treatment did not appear to blunt the reduction of CFU-GM (1.5-fold decrease) or CFU-GEMM (7.8-fold decrease) numbers in *Meis1* deleted mice compared to *MxCre/Meis1*^*-/+*^ mice, also treated with NAC, in myeloid biased media (Fig 14A). Using erythroid supportive conditions, numbers of large BFU-E (>16 colonies) also were not rescued by NAC administration in *MxCre/Meis*^*-/-*^ mice compared to *MxCre/Meis1*^*-/+*^ (12.8-fold decrease; Fig 14B).

**Fig 15:**
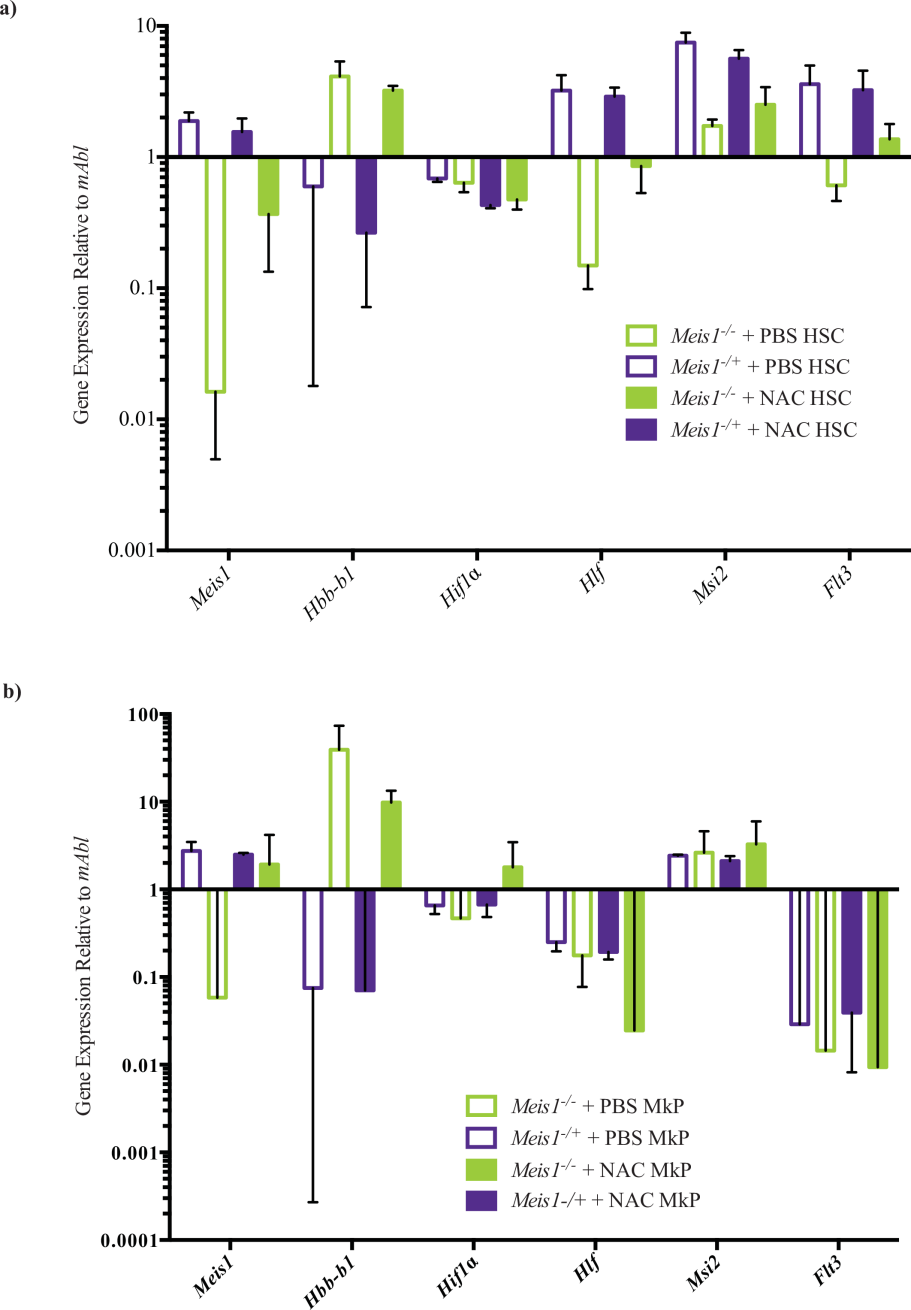
Gene expression changes as a result of NAC-treatment in sorted HSC and MkP populations. **a**) Gene expression changes as a result of loss of *Meis1* in the sorted HSC population treated with NAC or PBS. In PBS treated mice, there is a significant difference between *MxCre/Meis1*^*-/-*^ mice and *MxCre/Meis1*^*-/+*^ mice in the expression of *Meis1* (115-fold), *Hlf* (21-fold), *Msi2* (4.3-fold) and *Hbb-b1*(8-fold gain). While differences persist with NAC treatment the magnitude is blunted compared to PBS treated mice: *Meis1* (4-fold), *Hlf* (4-fold), *Msi2* (2-fold) and *Hbb-b1*(12-fold gain). **b**) Very few significant gene expression changes were found between *MxCre/Meis1*^*-/-*^ and *MxCre/Meis1*^*-/+*^ treated MkP cells. In PBS treated mice, there was a 47-fold drop in *Meis1* expression and in NAC treated mice, a 8-fold loss of *Hlf* and 140-fold gain in *Hbb-b1* expression.

We additionally sorted HSCs (LSKCD150^+^CD48^-^) and MkPs from *MxCre/Meis*^*-/-*^ and *MxCre/Meis1*^*-/+*^ PBS and NAC treated mice 5 days after the final treatment to examine if NAC treatment had any impact on a subset of genes previously identified as, altered upon *Meis1* deletion (Table 4) as well to examine if any key expression changes may be relevant in the MkP population. Quantiative RT-PCR results (Fig 15A) confirmed that loss of *Meis1* in HSCs results in reduction of expression of *Hlf* and *Msi2*, and enhanced expression of *Hbb-b1*. Neither *Msi2* nor *Hlf* expression was significantly altered in the MkP population (Fig 15B), suggesting *Meis1* regulation of these genes is exclusive to HSC-enriched cell populations as opposed to *Meis1*-expressing cells in general. In the presence of NAC, there were still reductions in *Hlf* and *Msi2* expression in the absence of *Meis1*, although the magnitude of this change was blunted compared to PBS treated *Meis1* deleted mice (*Hlf*, 3-fold reduction in NAC treated Meis1 deleted compared to 21-fold reduction in PBS control; *Msi2*, 2-fold reduction in NAC treated Meis1 deleted compared to 4.3-fold reduction in PBS control).

## Discussion

The primary goal of the studies described was to achieve a better understanding of the roles *Meis1* may play in adult hematopoiesis. To this end we have exploited mouse models in which *Meis1* can be conditionally deleted using two different Cre induction strategies. Four key findings emerge from these studies. First we corroborate previous findings that *Meis1* is essential for the homeostatic maintenance and regenerative capacity of adult HSCs and further quantify the nature of this deficit in the absence of *Meis1*. Second, we provide new evidence that *Meis1* is also essential in the early steps of megakaryocytic and erythroid pathways, particularly in the expansion of committed progenitors with proliferative potential. Third, our results of gene expression analyses support *Hlf* and *Msi2* as novel putative effectors of *Meis1*’s activity. Fourth, the impact of *Meis1* deletion can be blunted using scavengers of reactive oxygen species. This is in the context of our model system where *Meis1* does not appear to grossly impact putative Meis1 effector *Hif1α*, suggesting that ROS is important for *Meis1* function through alternate ROS pathways.

### *Meis1* is required for HSC maintenance and self-renewal

We found that *Meis1* is required for the HSC maintenance by phenotypic and functional lines of evidence. In the absence of *Meis1*, there was a 5-fold reduction in the numbers of phenotypically defined HSCs (LSKCD150^+^CD48^-^) in the *MxCre* model that correlates to a 10.7-fold reduction in functionally defined HSCs by long-term repopulating assays. This is supported by a 19-fold reduction in HSCs in long-term repopulating assays in the *ERTCre* model in the absence of *Meis1* compared to heterozygous controls. In a serial transplantation assay we show that *Meis1*^*-/-*^ HSCs are almost devoid of self-renewal capacity as they fail to contribute to hematopoietic reconstitution in secondary recipients. This is in contrast to *Meis1*^*-/+*^ cells that are able to sustain long-term repopulation in secondary recipients, representing, at minimum, a 25-fold reduction in self-renewal capacity between *Meis1*^*-/-*^ and *Meis1*^*-/+*^ HSCs. We additionally show this deficit to be largely cell intrinsic as there is a 6.3-fold drop in HSC frequency in *Meis1*^-/-^ cells compared to *Meis1*^*-/+*^ when transplanted into wild-type recipients prior to induction of allele deletion.

Overall our findings are consistent with two recently published studies using the same *Meis1* conditional knockout allele [6, 7]. While Unnisa *et al*. crossed mice onto the same *Rosa26ERTCre* strain as some of our studies, Kocobas *et al*. used the *SclERTCre* mouse where Cre expression is driven from the *Scl* promoter which is expressed in primitive hematopoietic, erythroid and megakaryocytic cells as well as in endothelium and specific neural tissues [29, 30]. The studies of Unnisa *et al*. and Kocabas *et al*. documented a loss of phenotypically and functionally defined HSC similar to our studies. Our additional use of limiting dilution assays add further resolution to the magnitude of of the effect of *Meis1* deletion on HSC frequency and self-renewal.

Unnisa *et al*. and Kocabas *et al*. also document increased proportions of *Meis1*^*-/-*^ cells in G_1_+S-G^2^-M using Hoechst/Pyronin in their respective HSC-enriched compartments (LSKCD150^+^CD48^-^ and LSKFlk2^-^CD34^-^, respectively). Despite the evidence from our GSEA of implicating *Meis1* in genes involved in mitosis, we were unable to document cell cycle perturbations in our studies using *in vivo* or *in vitro* BrdU (data not shown) possibly due to limitations in cell number and sensitivity of this method. Experimental variables relating to the use of controls could also be a factor in cell cycle results observed in these different studies. Whereas we utilized treated *Meis1*^*-/+*^ mice as controls, Unnisa *et al* used both non-Cre expressing and Cre expressing *Meis1*^*fl/+*^ mice as controls, whereas Kocabas *et al*. also included *Meis1*^*+/+*^ and *Meis1*^*fl/+*^ non-Cre expressing and Cre expressing mice as controls. Even in the absence of loxP sites, Cre expression in mammalian cells leads to reduced cell proliferation and accumulation in G_2_/M in a Cre-dose dependent manner [31]. It is thus possible that differences in cell cycling in the Kocabas *et al*. and Unnisa *et al*. studies may be due to a lack of Cre-mediated cell cycle depression in a significant proportion of their controls as opposed to a genuine influence of loss of *Meis1*. While the major finding of a loss of HSC potential remains consistent among the three studies, certain key differences highlight the need for further study.

### *Meis1* is required for megakaryopoiesis and erythropoiesis in the adult

Conflicting lines of evidence in several different experimental models exist for a role for *Meis1* in erythropoiesis and megakaryopoieis. During mouse development, in the absence of *Meis1*, megakaryocytes fail to form and erythroid colony numbers are reduced in the fetal liver [4, 5]. In zebrafish, *Meis1* knockdown results in loss of erythroid progenitors in mature fish [32], although a recent study using embryonic stem cell derived hematopoietic cells suggests *Meis1* represses erythroid development at the MEP stage in favour of megakaryocyte development [33]. In this work, we employed functional assays for megakaryocyte and erythroid potential and use a stress-erythropoiesis model to further expand requirement for *Meis1* in the expansion of erythroid progenitors.

Our studies show a critical and functional role in adult megakaryo- and erythropoiesis. We found a loss in platelets in both our models as well as reductions in RBC numbers in the *ERTCre* model. Reduced numbers of terminally differentiated mature cell types was supported by a 9-fold reduction in large CFU-Mk colonies and 11-fold loss of phenotypically defined megakaryocyte progenitors. There was a 4-fold reduction in BFU-E colonies in the BM of *MxCre/Meis*^*-/-*^ cells compared to *Meis1*^*-/+*^ controls. The loss of erythroid potential in the absence of *Meis1* was exacerbated in the PHZ model of haemolytic anemia and stress erythropoiesis where the proliferative potential of *Meis1*^*-/-*^ erythroid progenitors is severely blunted.

In the *ERTCre*-model, we found a loss of mature megakaryocytes histologically in the BM of moribund mice that was reflected in a loss of platelet numbers in the PB of these mice. Interestingly, in the *MxCre* model, no such loss of mature megakaryocytes was seen histologically, although there was a loss of phenotypically defined megakaryocytic progenitors, primitive colony-forming cells and mature platelets. This phenomenon of altered progenitor number without an apparent reduction of the mature cell type is reminiscent of the role of TGF-β1 in the erythroid population where expression serves to stimulate differentiation [34]. Although primitive cell number and mature megakaryocyte platelet output is reduced, intermediary mature megakaryocyte number may appear grossly normal. Histological evaluation at a later time point or following forced expansion in response to stress may reveal a loss of mature megakaryocytes in *MxCre/Meis1*^*-/-*^ mice, similar to *ERTCre/Meis1*^*-/-*^ mice.

Recent work using tracing techniques suggests that in the steady state, hematopoiesis is maintained by large numbers of lineage-restricted progenitors [35]. Our findings of minimal morbidity but alterations in PB composition at early time points in both the *ERTCre/Meis1* and *MxCre/Meis1* mice, the ability to detect CFCs of myeloid, erythroid and mixed lineage from *Meis1*^*-/-*^ cells, in addition to a failure of BFU-E expansion in the setting of PHZ, may support a model whereby *Meis1* expression is required for proliferation of erythroid and megakaryocytic progenitors but is dispensable for differentiation along these lineages and is dispensable for expansion of such lineage-restricted progenitors that provide the bulk of functional cell output. Use of cell tracing techniques in these conditional *Meis1* deletion models are warranted to determine if the deficit in these lineages is due solely to a lack of upstream HSC or if lack of proliferation of megakaryocytic and erythroid progenitors plays a more predominant role in this phenotype.

### *Hlf* and *Msi2* are putative effectors of *Meis1* function in adult hematopoiesis

Overall, we identified loss of expression of 4 genes (*Hlf*, *Msi2*, *Olfr4-2* and *Vamp5*) and gain of expression of 4 genes (*Il18r1*, *Tyrobp*, *Lilrb4*, and *Hbb-b1*) in response to loss of *Meis1* that met both criteria of a >90% confidence interval and >2-fold change. *Meis1* has previously been shown to be a transcriptional activator, thus, up-regulated genes in this model do not likely represent direct Meis1 targets. Up-regulated genes in our data set are primarily implicated in cell maturation and immune response, and are likely reflective of the loss of potential in the population studied and expression of Cre recombinase.

Our Affymetrix analysis and RT-Q-PCR identified *Hlf* and *Msi2* as a possible effector of *Meis1* function in an HSC-enriched population in the *MxCre/Meis1* model, consistent with findings by Ariki *et al*. using the same model system. *HLF* has been previously implicated in leukemia as a fusion with *E2A* in B-precursor ALL [23] as well as direct target of *Meis1* in Hox-mediated transformation [27]. E2A-HLF translocations are also thought to mediate leukemogenesis via Hox-independent mechanisms [36], highlighting Hox-independent functions for *Meis1*-mediated leukemogenesis. *Hlf* is differentially methylated and silenced through differentiation [37] and expression is enriched in the HSC population (www.immgen.com), supporting a possible role in HSC maintenance. Evidence for direct binding and regulation of *Hlf* by *Meis1* in leukemia [27] and our work suggests that *Meis1* may mediate possible *Hlf* function in the HSCs. More recently, Roychoudhury *et al*. [38] found that HLF overexpression could rescue colony forming deficits in the absence of *Meis1* in MLL-fusion models of AML, supporting *Hlf* as a key downstream mediator of *Meis1* function in both normal hematopoietic and leukemic processes.

Our screen for *Meis1* targets also identified *Msi2*, similarly to Ariki *et al*. *Msi2* has been implicated in the maintenance of HSC repopulation potential [39] and AML prognosis [40]. *Msi2* was also identified as part of a gene signature characterized by persistent *Vp16-Meis* transactivation in *Hox* models of leukemia [24]. The gene was first characterized in *D*. *melagnoster* as a regulator of asymmetric cell fate and may be involved in maintaining HSC quiescence via regulation of *Hes1* expression, a downstream effector of Notch signalling. Regulation of *Msi2* may be partially responsible for the deficits in BFU-E expansion in our studies as *Msi2* selectively expressed in cycling LT-HSC and down-regulated with differentiation, with the exception of re-expression in the BFU-E [39].

Regulation of *Hlf* and *Msi* by *Meis1* is also supported by ChIP-Seq experiments in our lab using a *Hox*-*Meis1* overexpression model (Yung *et al*., unpublished data) that demonstrates MEIS1 binding in the body of *Msi2* and in the transcription start side of *Hlf*. We examined changes of *Hlf* and *Msi* expression in highly purified populations of HSCs (LSKCD150^+^CD48^-^) and MkPs. These genes were consistently changed with loss of *Meis1*-expression in the HSC population, but not the MkP population, supporting a key role for *Hlf* and *Msi2* as effectors of *Meis1* function in the HSC compartment.

### Regulation of ROS plays a role in *Meis1* function

Although ROS and relative hypoxia had been previously implicated in HSC function (reviewed in [41]**)**, it was while examining the metabolic state of LT-HSC (as defined as Lin^-^Sca1^-^c-Kit^+^CD34^-^Flk2^-^ in their studies), that Simsek *et al*. [28] first drew a link between ROS and *Meis1*. Their work showed that LT-HSC could be enriched on the basis of low metabolic activity and that this fraction was enriched for Hif1α protein, a mediator of adaptation to low oxygen environments. In addition, they highlighted a conserved MEIS1 binding site in the promoter of *Hif1α* and demonstrated regulation of *HIf1α* expression by MEIS1 through luciferase and shRNA experiments. A role for *Meis1* in hypoxia tolerance was a novel discovery and triggered subsequent work looking at *Hif1α* expression and ROS regulation by *Meis1* in the HSC compartment ([6, 7], this work).

Hif1α dimerization to Hif1β is thought to trigger gene expression programs that allow adaptation to hypoxic environments. These programs are hypothesized to protect against the DNA damage caused by high ROS levels in the hypoxic environment that lead to HSC apoptosis. This theory has been supported by studies using NAC as an ROS scavenger. NAC provides a source of glutathione as a substrate for degradation reactions of peroxide (H_2_O_2_) into O_2_ and H_2_O and thus reduces ROS levels following cellular treatment. Several studies have used restoration of HSC function with NAC treatment to infer ROS scavenging rescues the deficient phenotype [6, 7, 42]. This is problematic, however, as NAC activity is not isolated to this role. NAC has been shown to modify the activity of key cell signalling and cycle cascade members Raf-1, MEK and ERK1/2, independently of its effect on ROS scavenging to promote cell survival [43]. Thus it is difficult to interpret if rescue using NAC is a result of reduction of ROS species or stimulation of the survival and cycling cascades influenced by Raf-1, MEK and ERK1/2.

Our studies show that treatment with the ROS scavenger N-acetyl-L-cysteine (NAC) abolished any phenotypic differences between *MxCre/Meis1*^*-/-*^ and *MxCre/Meis1*^*-/+*^ mice in HSC and MkP-enriched populations. Our data and emerging evidence suggests ROS may be important in both these populations, however, we were unable, through multiple methods in both models systems, to detect any changes in *Hif1α* (or *Hif1β*) as reported by other studies [7, 28]. This may be due to methodological issues, our model systems or most likely a combination thereof. Similarly to Ariki *et al*., *Hif1α* expression was found to be unchanged in our HSC-enriched LSK population by Affymetrix, nor by directed RT-Q-PCR with loss of *Meis1* using the *MxCre/Meis1* model. This could be due to a technical problem in our studies or low levels of *bona-fide* HSC in the KSL population. Most LSK cells (~49/50) are not HSCs. Thus, lack of a difference between *Meis*^*-/+*^ and *Meis1*^*-/-*^ LSK cells does not preclude the possibility of expression differences in the HSC compartment that we are not able to detect in this design. Arguing against this, however, our directed RT-Q-PCR in the more highly enriched LSKCD150^+^CD48^-^ population, also did not reveal changes in *Hif1α* expression.

Interplay between ROS, the niche, cell signalling and differentiation may also be of importance in megakaryocyte commitment and differentiation. Recent evidence suggests that ROS may be a signal for megakaryocyte lineage commitment and proliferation from the HSC progenitor through activation of the MEK-ERK1/2 pathway (reviewed in [44]). In our study with *in vivo* NAC treatment, NAC treatment abolished phenotypic HSCs and MkP differences between *Meis1*^*-/+*^ and *Meis*^*-/-*^ mice compared to PBS treated controls. As both ROS and NAC treatment stimulate MEK-ERK1/2 in the MkP, it is difficult to infer whether *Meis1* regulation of *Hif1α* plays any role in the MkP homeostasis. In our analysis of gene expression in the MkP population, no significant differences in *Hif1α* or *Hif2α* expression was found with either PBS or NAC-treatment between *MxCre/Meis1*^*-/-*^ and *MxCre/Meis1*^*-/+*^mice.

Interestingly, Roychoudhury *et al*.’s [38] recent work in mouse models and human samples of AML were also unable to detect changes in expression levels of *Hif1α* but supported a role for ROS in the pathogenesis of *Meis1*-deletion using an alternate model of ROS reduction, dichloroacetate. Overall, our data supports a role for ROS in the pathogenesis of *Meis1* deletion but suggests *Hif1α* is not a universal factor and other hypoxia-responsive pathways may be responsible for the bulk of this effect. Further work is needed to more clearly define these pathways.

## Conclusions

There is mounting evidence that *Meis1* is crucial for HSC and select progenitor compartments. Our work quantified HSC deficits in response to *Meis1* as well as highlighted key roles in the erythroid and megakaryocytic compartments. While our model systems support a role for *Meis1* in regulation of ROS, we were unable to attribute this to regulation of *Hif1α* as has been shown by others. We are able to support a role for *Hlf* and furthermore suggest *Msi2* also warrants further investigation. We would suggest that possibly the most intriguing implications of conditional *Meis1*-deletion is in how the model systems can be exploited to further refine our understanding of both normal hematopoietic and leukemic hierarchies.

## Supporting Information

S1 File **Table A: Dilution, clone and source of antibodies for FACS phenotyping and cell sorting. Table B: References for Sorting Gates.** All samples are first gated on SSC-A/FSC-A for size and complexity, DAPI for viability and FSC-A/FSC-H for singlets. Lineage refers to the lineage cocktail referred to as Lin- (IL7R is excluded in the CLP stain). **Table C: Probes for Southern blots and primer sets for Q-PCR detection of *Meis1***^***fl/fl***^ **genomic collapse. Table D: Primetime Q-RT-PCR assays**. **Table E: Genes differentially expressed with deletion of *Meis1* as determined by Affymetrix analysis within a 90% adjusted confidence interval**. **Fig A:** Southern blot of loxP targeted *Meis1* tissues using various induction schemes for *in vivo* induction of Cre expression in *MxCre/Meis1*^*tg*^ and *ERTCre/Meis1*^*tg*^ mice. Tissue-derived DNA was digested with BamHI and probed in the region anchored in loxP-B. Percent deletion of the floxed *Meis1* allele was calculated using densitometry software (ImageQuant, GE). One of each *Meis1*^*+/fl*^ and *MxCre/Meis1*^*+/fl*^ mice were used in the first 3 injection induction experiment whereas a total of 6 *MxCre/Meis1*^*+/+*^, *MxCre/Meis1*^*fl/+*^ and *MxCre/Meis1*^*fl/fl*^ mice were used in the second attempt. 2 *ERTCre/Meis1*^*fl/fl*^ mice were compared to a *ERTCre/Meis1*^*fl/+*^and *Meis1*^*fl/fl*^ mouse. Mice were assessed 2 days after the last IP injection. **Fig B:** Sequencing results confirming introduction of premature stop codon in exon 9 of Meis1fl with expression of Cre recombinase. cDNA from ERTCre/Meis1-/+, ERTCre/Meis1-/-, and ERTCre/Meis1+/+ splenocytes was amplified using primers in exon 7 and exon 11 and cloned into the TOPO-TA vector for sequencing. Sequencing of the clones confirmed generation of the predicted transcript with a premature stop codon in exon 9 following Cre recombinase expression.
(DOCX)
