# Supplementary material for: Meis1 Is Required for Adult Mouse Erythropoiesis, Megakaryopoiesis and Hematopoietic Stem Cell Expansion
Source: PLoS One. 2016 Mar 17;11(3):e0151584. doi: 10.1371/journal.pone.0151584 (PMC4795694; doi:10.1371/journal.pone.0151584)
Supplement: S1 File — Table A: Dilution, clone and source of antibodies for FACS phenotyping and cell sorting. Table B: References for Sorting Gates. All samples are first gated on SSC-A/FSC-A for size and complexity, DAPI for viability and FSC-A/FSC-H for singlets. Lineage refers to the lineage cocktail referred to as Lin- (IL7R is excluded in the CLP stain). Table C: Probes for Southern blots and primer sets for Q-PCR detection of Meis1fl/fl genomic collapse. Table D: Primetime Q-RT-PCR assays. Table E: Genes differentially expressed with deletion of Meis1 as determined by Affymetrix analysis within a 90% adjusted confidence interval. Fig A: Southern blot of loxP targeted Meis1 tissues using various induction schemes for in vivo induction of Cre expression in MxCre/Meis1tg and ERTCre/Meis1tg mice. Tissue-derived DNA was digested with BamHI and probed in the region anchored in loxP-B. Percent deletion of the floxed Meis1 allele was calculated using densitometry software (ImageQuant, GE). One of each Meis1+/fl and MxCre/Meis1+/fl mice were used in the first 3 injection induction experiment whereas a total of 6 MxCre/Meis1+/+, MxCre/Meis1fl/+ and MxCre/Meis1fl/fl mice were used in the second attempt. 2 ERTCre/Meis1fl/fl mice were compared to a ERTCre/Meis1fl/+and Meis1fl/fl mouse. Mice were assessed 2 days after the last IP injection. Fig B: Sequencing results confirming introduction of premature stop codon in exon 9 of Meis1fl with expression of Cre recombinase. cDNA from ERTCre/Meis1-/+, ERTCre/Meis1-/-, and ERTCre/Meis1+/+ splenocytes was amplified using primers in exon 7 and exon 11 and cloned into the TOPO-TA vector for sequencing. Sequencing of the clones confirmed generation of the predicted transcript with a premature stop codon in exon 9 following Cre recombinase expression. (DOCX) [file pone.0151584.s001.docx]

S1 Table A: Name, clone, dilution and supplier of antibodies for FACS phenotyping and cell sorting

| **PURPOSE** | **ANTIBODY** | **CLONE** | **DILUTION (FINAL)** | **SUPPLIER** |
| --- | --- | --- | --- | --- |
| Donor cell and lineage distribution of mouse peripheral blood | CD45.2-APC | 104 | 1:1000 | BD Pharmingen |
|  | CD4-PE | L3T4 | 1:1600 | eBioscience |
|  | CD8-PE | Ly-2 | 1:1600 | eBioScience |
|  | B220-PE | RA3-6B2 | 1:2000 | BD Pharmingen |
|  | B220-APC-Cy7 | RA3-6B2 | 1:2000 | BD Pharmingen |
|  | Gr1-APC-Cy7 | RB6-8C5 | 1:6000 | BD Pharmingen |
|  | Mac1-APC-Cy7 | M1/70 | 1:5000 | BD Pharmingen |
| ESLAM HSC for expression profiling [1] | CD45-FITC | 30-F11 | 1:100 | BioLegend |
|  | EPCR-PE | RMEPCR1560 | 1:100 | STEMCELL Technologies |
|  | CD48-APC | HM48-1 | 1:100 | BioLegend |
|  | CD150-biotin | TC15-12F12.2 | 1:100 | BioLegend |
|  | SA-PE-TxRed | Streptavidin | 1:400 | BD Pharmingen |
| CD150 HSC for phenotyping [2] | Lineage-PerCP-Cy5.5 | See below | See below | See below |
|  | cKit-APC | 2B8 | 1:100 | BD Pharmingen |
|  | Sca-PE | E13-161.7 | 1:100 | BD Pharmingen |
|  | CD48-FITC | HM48-1 | 1:100 | BioLegend |
|  | CD150-PE-Cy7 | SLAM | 1:100 | BioLegend |
| LSK for Affymetrix expression analysis | Lineage-PerCP-Cy5.5 | See below | See below | See below |
|  | cKit-APC | 2B8 | 1:100 | BD Pharmingen |
|  | Sca-PE | E13-161.7 | 1:100 | BD Pharmingen |
| Myeloid progenitors for expression and phenotyping [3] | Lineage-PerCP-Cy5.5 | See below | See below | See below |
|  | Sca1-PE-Cy7 | E13-161.7 | 1:150 | BD Pharmingen |
|  | cKit-APC | 2B8 | 1:100 | BD Pharmingen |
|  | CD34-FITC | RAM34 | 1:400 | eBioscience |
|  | CD16/32-PE | 2.4G2 | 1:300 | BD Pharmingen |
| Common lymphoid progenitors (CLP) for expression and phenotyping [4] | Lineage-FITC | See below | See below | See below |
|  | cKit-APC | 2B8 | 1:100 | BD Pharmingen |
|  | Sca1-PE | E13-161.7 | 1:100 | BD Pharmingen |
|  | CD127-biotin | B12-1 | 1:100 | BD Pharmingen |
|  | SA-PerCP-Cy5.5 | Streptavidin | 1:100 | BD Pharmingen |
| Megakaryocyte progenitors for expression and phenotyping [5] | Lineage-PerCP-Cy5.5 | See below | See below | See below |
|  | Sca1-PE | E13-161.7 | 1:200 | BD Pharmingen |
|  | cKit-APC | 2B8 | 1:100 | BD Pharmingen |
|  | CD150-PE-Cy7 | SLAM | 1:100 | Biolegend |
|  | CD41-FITC | MWReg30 | 1:100 | BD Pharmingen |
| Mature megakaryocytes  [6] | CD41 (as per Nilsson lab) |  |  |  |
| Erythroblast maturation series for expression profiling *unlysed [7] | Ter119-PerCP-Cy5.5 | TER-119 | 1:150 | BD Pharmingen |
|  | CD71-PE | C2 | 1:100 | BD Pharmingen |
| Lineage-FITC for expression profiling and phenotyping of CLP | Gr1-FITC | RB6-8C5 | 1:2400 | BD Pharmingen |
|  | Ter119-FITC | TER-119 | 1:150 | BD Pharmingen |
|  | B220-FITC | RA3-6B2 | 1:600 | BD Pharmingen |
|  | CD3-FITC | L3T4 | 1:300 | BD Pharmingen |
|  | CD4-FITC | Ly-2 | 1:600 | BD Pharmingen |
|  | CD8a-FITC | 53-6.7 | 1:600 | BD Pharmingen |
| Lineage-PerCP-Cy5.5 for expression profiling and phenotyping of HSC and myeloid lineages | Gr1-PerCP-Cy5.5 | 1A8 | 1:2400 | BD Pharmingen |
|  | Ter119- PerCP-Cy5.5 | TER-119 | 1:150 | BD Pharmingen |
|  | B220- PerCP-Cy5.5 | RA3-6B2 | 1:600 | BD Pharmingen |
|  | CD3- PerCP-Cy5.5 | L3T4 | 1:300 | BD Pharmingen |
|  | CD4- PerCP-Cy5.5 | Ly-2 | 1:600 | BD Pharmingen |
|  | CD8a- PerCP-Cy5.5 | 53-6.7 | 1:600 | BD Pharmingen |
| Mature lymphoid for expression profiling | B220-APC-Cy7 | RA3-6B2 | 1:500 | BD Pharmingen |
|  | CD4-PE | L3T4 | 1:400 | eBiosciences |
|  | CD8-PE | Ly-2 | 1:400 | eBiosciences |
| Mature myeloid for expression profiling [8] | Gr1-APC-Cy7 | RB6-8C5 | 1:500 | BD Pharmingen |
|  | Mac1-FITC | M1/70 | 1:800 | BD Pharmingen |
| Apoptosis | AnnexinV-PE |  | kit instructions | BD Pharmingen |
| Cell Cycle | BrDU-APC |  | kit instructions | BD Pharmingen |

S1 Table B: References for Sorting Gates

All samples are first gated on SSC-A/FSC-A for size and complexity, DAPI for viability and FSC-A/FSC-H for singlets. Lineage refers to the lineage cocktail referred to as Lin- (IL7R is excluded in the CLP stain).

| Stain | Markers | Reference |
| --- | --- | --- |
| Lineage | Gr1, Mac1, Ter119, B220, CD3, CD8 (IL7R) | - |
| ESLAM HSC | CD45^mod^EPCR^+^CD48^-^CD150^+^ | Kent *et al.*, 2009 |
| CD150 HSC | Lin^-^cKit^+^Sca1^+^CD48^-^CD150^+^ | Kiel *et al*., 2005 |
| CMP | Lin^-^cKit^+^Sca1^-^CD16/32^low^CD34^+^ | Akashi *et al.*, 2000 |
| GMP | Lin^-^cKit^+^Sca1^-^CD16/32^hi^CD34^+^ | Akashi *et al.*, 2000 |
| MEP | Lin^-^cKit^+^Sca1^-^CD16/32^low^CD34^low^ | Akashi *et al.*, 2000 |
| MkP | Lin^-^cKit^+^Sca1^-^CD150^+^CD41^+^ | Pronk *et al.*, 2007 |
| CLP | Lin^-^IL7R^+^cKit^mid^Sca1^mid^ | Kondo *et al.*, 1997 |
| Immature Erythroblast | Ter119^+^CD71^hi^ | Socolovsky *et al.*, 2001 |
| Maturing Erythroblast | Ter119^+^CD71^mid^ | Socolovsky *et al.*, 2001 |
| Mature Erythroblast | Ter119^+^CD71^low^ | Socolovsky *et al.*, 2001 |
| Granulocyte Precursor | Gr1^+^Mac1^+^ | Song *et al*., 2005 |
| Granulocyte | Gr1^-^Mac1^+^ | Song *et al*., 2005 |
| Mature Megakaryocyte | CD41^+^SSC^hi^ | Heazlewood *et al.*, 2013 |

S1 Table C: Probes for Southern blots and primer sets for Q-PCR detection of *Meis1^fl/fl^* genomic collapse

| **Purpose** | **Probe** | **Position** | **Forward** | **Reverse** |
| --- | --- | --- | --- | --- |
| Southern Blot Analysis | Internal | 5’ of the 3’ LoxP site | 5’-gatttgatgctcttgcgaca-3’ | 5’-gaagttattaggtggatccaagct-3’ |
|  | External | 5’ of the 5’ LoxP site | 5’-ccgtggttctccaagtttgt-3’ | 5’- tccatctcaaaccccttcag-3’ |
|  | **Probe** | **Digest** | **WT hybridization** | **MUT allele hybridization** |
|  | Internal | HindIII | 7.4 kbp | 2.3 kbp |
|  | Internal | EcoRI | 3 kbp | 2.1 kpb/no band with Cre |
|  | External | BglII | 2.3 kbp | 2.4 kbp/3.8kbp with Cre |
| Cloning RT-PCR | Meis1 exon 7 – exon 11 |  | 5’-TCCACTCGTTCAGGAGGAAC-3’ | 5’-TGCTGACCGTCCATTACAAA-3’ |
| Deletion detection RT-PCR | NDF | Non-deleted floxed | 5’ - agcttcatttgaagttccctattg-3’ | 5’- tattaggtggatccaagcttcatt-3’ |
|  | DF | Deleted floxed | 5’- ctggactttctcctttagttggat-3’ | 5’- ggaacttcatcagtcaggtacata-3’ |
|  | Floxed CTL | Floxed (regardless of deletion) | 5’-tatgtacctgactgatgaagttcc -3’ | 5’- gcgtcacttggaaaagcaat-3’ |
|  | Exon 7 CTL | Endogenous CTL | 5’- ttggaatagagaccatgatgacac-3’ | 5’- gttatccccactgtgtgaagtatg-3’ |

**S1 Table D: Primetime Q-RT-PCR assays**

| **Primetime Assays** | **IDT Assay ID** |
| --- | --- |
| mABL1 | [Mm.PT.42.14158394](javascript:__doPostBack('ctl06$NameEditBtn_32076900','')) |
| mMeis1 | Mm.PT.42.14235881 |
| mMeis2 | Mm.PT.45.7421202 |
| mMeis3 | Mm.PT.42.9683604 |
| mGata1 | Mm.PT.45.10444529 |
| mGata2 | Mm.PT.45.13913016.g |
| mGata3 | Mm.PT.45.11120670.g |
| mPrep1 | Mm.PT.45.16285104 |
| mPrep2 | Mm.PT.45.14033370 |
| mNotch1 | Mm.PT.45.10390781 |
| mIL7r | Mm.PT.45.14297778 |
| mIL18r | Mm.PT.45.11896127 |
| mHbb-b1 | Mm.PT.42.10942091 |
| mHbb-b2 | Mm.PT.45.10154419 |
| mHlf | Mm.PT.45.15839543 |
| mMpo | Mm.PT.45.15839547 |
| mMsi2 | Mm.PT.45.17224408 |
| mPcgf5 | Mm.PT.45.10726269 |
| mSelp | Mm.PT.45.16240531 |
| mSenp8 | Mm.PT.45.12425187.g |
| mTyrobp | Mm.PT.45.11022459 |
| mVamp5 | Mm.PT.45.5620876 |
| mAurkb | Mm.PT.47.5154422 |
| mCcna2 | Mm.PT.47.1386893 |
| mCcnb2 | Mm.PT.47.17484650 |
| mCcnd1 | Mm.PT.47.12022381 |
| mClec12a | Mm.PT.47.8638109 |
| mDdx4 | Mm.PT.47.16947842 |
| mDgat1 | Mm.PT.47.12387218 |
| mE2f8 | Mm.PT.47.7043043 |
| mFlt3 | Mm.PT.47.10501150 |
| mGria3 | Mm.PT.47.14084255 |
| mGstm5 | Mm.PT.47.11429296 |
| mHes1 | Mm.PT.47.8454373.g |
| mHoxA6 | Mm.PT.47.13073650 |
| mPtpn5 | Mm.PT.47.10380131.g |
| mRassf4 | Mm.PT.47.15971865 |
| mSfpi1 | Mm.PT.47.7508177 |
| mSnx31 | Mm.PT.47.15894188 |
| mHIF1α | Mm.PT.47.8983770 |
| mHIF2α | Mm.PT.47.7593249 |
| mP16Ink4a | Mm.PT.47.9881334 |
| mP19Arf | Mm.PT.47.5632963 |

S1 Table E: Genes differentially expressed with deletion of *Meis1* as determined by Affymetrix analysis within a 90% adjusted confidence interval

| **Probe Name** | **Description** | **Adjusted t-Test** | **Fold decrease in *Meis1^-/-^*** |
| --- | --- | --- | --- |
| 6836829 | Dgat1 | 0.006761443 | 0.686689462 |
| 6817970 | Nt5dc2 | 0.01133132 | 0.70196703 |
| 6828741 | C1qtnf3 | 0.01148776 | 0.829957265 |
| 6790699 | Hlf | 0.01207463 | 3.180698125 |
| 6942692 | Tmem184a | 0.03904288 | 0.919683837 |
| 6835004 | Snx31 | 0.03916838 | 1.214778913 |
| 6790621 | Msi2 | 0.04223439 | 2.070406603 |
| 6871457 | Incenp | 0.04471138 | 0.867291904 |
| 6929828 | Nat8l | 0.04471138 | 0.791477701 |
| 6798334 | Adam6b | 0.05381157 | 1.562741704 |
| 6993465 | Endod1 | 0.05650069 | 0.808002529 |
| 6993472 | Fut4 | 0.05650069 | 0.671168355 |
| 6953607 | Hoxa6 | 0.05650069 | 0.835739062 |
| 6808173 | Irx2 | 0.05650069 | 0.903497659 |
| 6992172 | Dusp7 | 0.07083209 | 0.709573183 |
| 6769213 | Plk5 | 0.07433471 | 0.958082417 |
| 6769381 | D10Wsu102e | 0.07433471 | 0.799486601 |
| 6790046 | Evi2b | 0.07433471 | 0.754337234 |
| 6974010 | Ing1 | 0.07433471 | 0.795613559 |
| 6992436 | Ngp | 0.07433471 | 0.686477168 |
| 6967109 | Ptpn5 | 0.07433471 | 0.953819688 |
| 6820084 | Reep4 | 0.07433471 | 0.750008593 |
| 6992178 | Rrp9 | 0.07433471 | 0.876239425 |
| 6916748 | Slc2a1 | 0.07433471 | 0.904081689 |
| 6883184 | Slc2a10 | 0.07433471 | 0.85757217 |
| 6857885 | Srbd1 | 0.07433471 | 0.75571711 |
| 6894253 | Chrna4 | 0.07433471 | 1.044130276 |
| 6933084 | D930016D06Rik | 0.07433471 | 1.512825956 |
| 6782496 | Dbil5 | 0.07433471 | 1.221825464 |
| 6869635 | Entpd1 | 0.07433471 | 1.258515318 |
| 6900348 | Gstm5 | 0.07433471 | 1.529539288 |
| 6962759 | Kctd14 | 0.07433471 | 1.338515966 |
| 6964011 | Scnn1g | 0.07623116 | 0.878159125 |
| 6867632 | Cabp2 | 0.07905712 | 0.92302027 |
| 6748889 | Il18r1 | 0.07905712 | 0.487679618 |
| 6978369 | Mmp15 | 0.07905712 | 0.946099273 |
| 6805191 | Olfr263-ps1 | 0.07905712 | 3.401042566 |
| 6789360 | 2810408A11Rik | 0.08290535 | 0.859347092 |
| 6895915 | Bhlhe22 | 0.08290535 | 0.907053702 |
| 6925562 | Zbtb8b | 0.08290535 | 0.91087139 |
| 6965893 | Lypd4 | 0.08290535 | 1.055802188 |
| 6763295 | Ralgps2 | 0.08290535 | 1.363438361 |
| 6982267 | Wwc2 | 0.08290535 | 1.165781047 |
| 6846010 | Cd96 | 0.09373904 | 0.655477699 |
| 6995384 | Fam55b | 0.09373904 | 0.77994938 |
| 6920609 | Gja10 | 0.09373904 | 0.847973393 |
| 6969997 | Hbb-b1 | 0.09373904 | 0.205045826 |
| 6852144 | Lbh | 0.09373904 | 0.742217798 |
| 6855706 | Srf | 0.09373904 | 0.796670523 |
| 6883261 | Trp53rk | 0.09373904 | 0.68789235 |
| 6815523 | Naip5 | 0.09373904 | 1.459240896 |
| 6751349 | Dgkd | 0.09440419 | 1.271864279 |
| 6917813 | Asap3 | 0.09593448 | 0.956210778 |
| 6767782 | Lilrb4 | 0.09593448 | 0.217899404 |
| 6959584 | Tyrobp | 0.09593448 | 0.477594239 |
| 6858134 | Nrxn1 | 0.09593448 | 1.52807978 |
| 6959536 | Zfp30 | 0.09593448 | 1.163797528 |
| 6858910 | Ttc39c | 0.09641302 | 0.885682526 |
| 6802491 | 6430527G18Rik | 0.09826282 | 0.822903471 |
| 6969016 | 9930013L23Rik | 0.09826282 | 0.934908463 |
| 6775322 | C030046I01Rik | 0.09826282 | 0.914925308 |
| 6966490 | C230052I12Rik | 0.09826282 | 0.835897232 |
| 6972491 | Ccnd1 | 0.09826282 | 0.78631627 |
| 6910126 | Clca2 | 0.09826282 | 0.875813922 |
| 6818044 | D830044D21Rik | 0.09826282 | 0.881678297 |
| 6873363 | Fgf8 | 0.09826282 | 0.839396182 |
| 6780332 | Gabrb2 | 0.09826282 | 0.744622975 |
| 6927253 | Gabrd | 0.09826282 | 0.903307584 |
| 6785213 | Galr2 | 0.09826282 | 0.817732162 |
| 6964600 | Gpr26 | 0.09826282 | 0.804403471 |
| 6981099 | Ido1 | 0.09826282 | 0.920582657 |
| 6838716 | Itgb7 | 0.09826282 | 0.861326652 |
| 6789197 | Ntn1 | 0.09826282 | 0.873434537 |
| 6863467 | Osbpl1a | 0.09826282 | 0.811192447 |
| 6751264 | Psmd1 | 0.09826282 | 0.903584256 |
| 6917963 | Rap1gap | 0.09826282 | 0.922605325 |
| 6983163 | Rfxank | 0.09826282 | 0.922160403 |
| 6799645 | Rps7 | 0.09826282 | 0.800539592 |
| 6801636 | Rtn1 | 0.09826282 | 0.931952816 |
| 6865957 | Slc6a7 | 0.09826282 | 0.923006603 |
| 6841739 | Tomm70a | 0.09826282 | 0.708964521 |
| 6977523 | Anapc10 | 0.09826282 | 1.102988954 |
| 6775441 | Atcay | 0.09826282 | 1.093399049 |
| 6937253 | Fam53a | 0.09826282 | 1.289113157 |
| 6943142 | Flt3 | 0.09826282 | 1.510209021 |
| 6980568 | Gas6 | 0.09826282 | 1.076483534 |
| 6791302 | Gjd3 | 0.09826282 | 1.212372281 |
| 6866800 | Katnal2 | 0.09826282 | 1.120357182 |
| 6755237 | Kcnj10 | 0.09826282 | 1.080823042 |
| 6838695 | Krt78 | 0.09826282 | 1.093853513 |
| 6936930 | Lmbr1 | 0.09826282 | 1.268653264 |
| 6818858 | Mudeng | 0.09826282 | 1.298148084 |
| 6818523 | Ptger2 | 0.09826282 | 1.153822817 |
| 6815382 | Rgnef | 0.09826282 | 1.080829635 |
| 6966041 | Shkbp1 | 0.09826282 | 1.039448805 |
| 6799842 | Slc26a4 | 0.09826282 | 1.065860955 |
| 6871139 | Mtvr2 | 0.09826282 | 1.12901198 |
| 6864444 | Stard4 | 0.09826282 | 1.188663435 |
| 6824195 | Txndc16 | 0.09826282 | 1.185122457 |
| 6954615 | Vamp5 | 0.09826282 | 5.77675678 |
| 6807209 | Zfp346 | 0.09826282 | 1.258522896 |
| 6872206 | 1700028P14Rik | 0.09871875 | 0.939795599 |
| 6867593 | 1810055G02Rik | 0.09871875 | 0.858117949 |
| 6969429 | 4632434I11Rik | 0.09871875 | 0.699203711 |
| 6899308 | 4933434E20Rik | 0.09871875 | 0.886234308 |
| 6875832 | Adamts13 | 0.09871875 | 0.949246684 |
| 6968126 | Arrdc4 | 0.09871875 | 0.789476573 |
| 6784765 | Axin2 | 0.09871875 | 0.909899641 |
| 6784329 | BC030867 | 0.09871875 | 0.859150792 |
| 6790199 | Ccl1 | 0.09871875 | 0.852827886 |
| 6773485 | Cdc40 | 0.09871875 | 0.644957771 |
| 6950137 | Clec12a | 0.09871875 | 0.55226552 |
| 6748695 | Cnga3 | 0.09871875 | 0.848009481 |
| 7016421 | Cul4b | 0.09871875 | 0.756217809 |
| 6861751 | D18Ertd653e | 0.09871875 | 0.859290586 |
| 6892580 | D630003M21Rik | 0.09871875 | 0.972298247 |
| 6871627 | Dtx4 | 0.09871875 | 0.920747116 |
| 6959674 | Gm6725 | 0.09871875 | 0.680986794 |
| 6935273 | Eif3b | 0.09871875 | 0.666257578 |
| 6953126 | Fam131b | 0.09871875 | 0.838222299 |
| 6947394 | Mogs | 0.09871875 | 0.884365136 |
| 6934506 | Glt1d1 | 0.09871875 | 0.893948355 |
| 6941215 | Gltp | 0.09871875 | 0.880693101 |
| 6840400 | Hes1 | 0.09871875 | 0.918382136 |
| 6762452 | Igfn1 | 0.09871875 | 0.918880257 |
| 6784845 | Kcnj2 | 0.09871875 | 0.869945205 |
| 6823849 | Mapk8 | 0.09871875 | 0.689086426 |
| 6829297 | March11 | 0.09871875 | 0.865598668 |
| 6850723 | Mea1 | 0.09871875 | 0.810454696 |
| 6876010 | Med27 | 0.09871875 | 0.816689095 |
| 6993272 | Mmp1b | 0.09871875 | 0.872917557 |
| 6850661 | Mrpl14 | 0.09871875 | 0.782647265 |
| 6943387 | N4bp2l1 | 0.09871875 | 0.789798996 |
| 6911925 | Nbn | 0.09871875 | 0.719175098 |
| 6804893 | Nid1 | 0.09871875 | 0.918265128 |
| 6832332 | Nup50 | 0.09871875 | 0.795160585 |
| 6850345 | Olfr130 | 0.09871875 | 0.917081849 |
| 6958995 | Opa3 | 0.09871875 | 0.808539415 |
| 6919095 | Pank4 | 0.09871875 | 0.869308084 |
| 6991358 | Plscr1 | 0.09871875 | 0.858824362 |
| 6924882 | Ptprf | 0.09871875 | 0.954875573 |
| 6791534 | Pyy | 0.09871875 | 0.82370519 |
| 6956765 | Rassf4 | 0.09871875 | 0.628405716 |
| 6789789 | Rph3al | 0.09871875 | 0.895808026 |
| 6797572 | Serpina5 | 0.09871875 | 0.823835983 |
| 6762423 | Shisa4 | 0.09871875 | 0.888228821 |
| 6768323 | Slc25a16 | 0.09871875 | 0.888569833 |
| 6978937 | Sntb2 | 0.09871875 | 0.87858046 |
| 6902192 | Ssx2ip | 0.09871875 | 0.966105003 |
| 6893057 | Sulf2 | 0.09871875 | 0.927836986 |
| 6837318 | Tob2 | 0.09871875 | 0.891491411 |
| 6971410 | Prss53 | 0.09871875 | 0.733547615 |
| 6768898 | Vpreb3 | 0.09871875 | 0.866078032 |
| 6888928 | Accs | 0.09871875 | 1.100261389 |
| 6972149 | BC066028 | 0.09871875 | 1.126072063 |
| 6864520 | Brd8 | 0.09871875 | 1.137338966 |
| 6968772 | Cib1 | 0.09871875 | 1.279751569 |
| 6845159 | Fbxo45 | 0.09871875 | 1.099376831 |
| 6934855 | Gatsl2 | 0.09871875 | 1.407191182 |
| 6961012 | Magel2 | 0.09871875 | 1.155860681 |
| 6907869 | Mov10 | 0.09871875 | 1.303027043 |
| 6970037 | Olfr649 | 0.09871875 | 1.128090383 |
| 6955066 | Paip2b | 0.09871875 | 1.161642402 |
| 6957178 | Plekhg6 | 0.09871875 | 1.048243756 |
| 6857834 | Prepl | 0.09871875 | 1.156735331 |
| 6854460 | Rab11fip3 | 0.09871875 | 1.075945323 |
| 6866862 | Setbp1 | 0.09871875 | 1.044104365 |
| 6762355 | Tmem183a | 0.09871875 | 1.227058832 |
| 6859415 | Zfp397 | 0.09871875 | 1.286213235 |
| 6862627 | Zfp516 | 0.09871875 | 1.940285837 |
| 6753091 | Elk4 | 0.09986983 | 1.358886916 |

**S1 Fig A:** Southern blot of loxP targeted *Meis1* tissues using various induction schemes for in vivo induction of Cre expression in *MxCre/Meis1^tg^* and *ERTCre/Meis1^tg^* mice. Tissue-derived DNA was digested with BamHI and probed in the region anchored in loxP-B. Percent deletion of the floxed *Meis1* allele was calculated using densitometry software (ImageQuant, GE). One of each *Meis1^+/fl^* and *MxCre/Meis1^+/fl^* mice were used in the first 3 injection induction experiment whereas a total of 6 *MxCre/Meis1^+/+^, MxCre/Meis1^fl/+^* and *MxCre/Meis1^fl/fl^* mice were used in the second attempt. 2 *ERTCre/Meis1^fl/fl^* mice were compared to a *ERTCre/Meis1^fl/+^*and *Meis1^fl/fl^* mouse. Mice were assessed 2 days after the last IP injection.

**S1 Fig B**: Sequencing results confirming introduction of premature stop codon in exon 9 of Meis1fl with expression of Cre recombinase. cDNA from ERTCre/Meis1-/+, ERTCre/Meis1-/-, and ERTCre/Meis1+/+ splenocytes was amplified using primers in exon 7 and exon 11 and cloned into the TOPO-TA vector for sequencing. Sequencing of the clones confirmed generation of the predicted transcript with a premature stop codon in exon 9 following Cre recombinase expression.

**Supplemental References**

1. Kent DG, Copley MR, Benz C, Wohrer S, Dykstra BJ, Ma E, et al. Prospective isolation and molecular characterization of hematopoietic stem cells with durable self-renewal potential. Blood. 2009 Jun 18;113(25):6342–50.

2. Kiel MJ, Yilmaz OH, Iwashita T, Yilmaz OH, Terhorst C, Morrison SJ. SLAM family receptors distinguish hematopoietic stem and progenitor cells and reveal endothelial niches for stem cells. Cell. 2005 Jul 1;121(7):1109–21.

3. Akashi K, Traver D, Miyamoto T, Weissman IL. A clonogenic common myeloid progenitor that gives rise to all myeloid lineages. Nature. 2000 Mar 9;404:193–7.

4. Kondo M, Weissman IL, Akashi K. Identification of clonogenic common lymphoid progenitors in mouse bone marrow. Cell. 1997 Nov 28;91(5):661–72.

5. Pronk CJH, Rossi DJ, Månsson R, Attema JL, Norddahl GL, Chan CKF, et al. Elucidation of the phenotypic, functional, and molecular topography of a myeloerythroid progenitor cell hierarchy. Cell Stem Cell. 2007 Oct 11;1(4):428–42.

6. Heazlewood SY, Neaves RJ, Williams B, Haylock DN, Adams TE, Nilsson SK. Megakaryocytes co-localise with hemopoietic stem cells and release cytokines that up-regulate stem cell proliferation. Stem Cell Res. 2013 May 28;11(2):782–92.

7. Socolovsky M, Nam H, Fleming MD, Haase VH, Brugnara C, Lodish HF. Ineffective erythropoiesis in Stat5a(-/-)5b(-/-) mice due to decreased survival of early erythroblasts. Blood. 2001 Dec 1;98(12):3261–73.

8. Song X, Krelin Y, Dvorkin T, Bjorkdahl O, Segal S, Dinarello CA, et al. CD11b+/Gr-1+ immature myeloid cells mediate suppression of T cells in mice bearing tumors of IL-1beta-secreting cells. J Immunol. 2005 Dec 15;175(12):8200–8.
